# Unveiling the intensity–ambiguity relationships among affective and lexico-semantic variables in Chinese characters and the character–word relationships in Chinese two-character words

**DOI:** 10.3758/s13428-025-02753-9

**Published:** 2025-07-15

**Authors:** Xi Cheng, Chi-Shing Tse, Yuen-Lai Chan, Kai Yan Lau, Yen Na Yum

**Affiliations:** 1 Department of Educational Psychology, The Chinese University of Hong Kong, Hong Kong, China; 2 Centre for Learning Sciences and Technologies, The Chinese University of Hong Kong, Hong Kong, China; 3 Department of Psychology, Lingnan University, Hong Kong, Hong Kong, China; 4 Department of Chinese and Bilingual Studies, The Hong Kong Polytechnic University, Hong Kong, China; 5 Department of Special Education and Counselling, The Education University of Hong Kong, Hong Kong, China

**Keywords:** Chinese Characters, Megastudy, Norming, Valence

## Abstract

Understanding the lexical characteristics of Chinese characters is crucial given their extensive usage and unique logographic structure. In this study, we normed affective ratings (valence and arousal) for 3971 Chinese characters. We investigated the relationships between intensity (mean rating) and ambiguity (rating variability) of these affective variables, alongside additional lexico-semantic variables from Su et al., *Behavior Research Methods*, *55*(6), 2989–3008, (2022). Drawing on lexical data from 25,281 two-character words available in the Chinese Lexicon Project (Tse et al., *Behavior Research Methods*, *49*(4), 1503–1519, 2017, *Behavior Research Methods*, *55*(8), 4382–4402, 2023; Chan & Tse, *Behavior Research Methods*, *56*(7), 7574–7601, 2024), we further explored cross-level relationships between character-level and word-level variables. Multiple regression analyses controlling for various lexical variables revealed several noteworthy patterns. First, we identified a quadratic valence–arousal relationship, such that characters with extreme valence ratings (either highly positive or highly negative) elicited higher arousal compared to neutral characters. This relationship was moderated by arousal ambiguity, partially consistent with previous findings (Brainerd et al. *Journal of Experimental Psychology: General*, *150*(8), 1476–1499, 2021a), Second, we observed consistent quadratic intensity–ambiguity relationships across all variables, supporting the quadratic law proposed by Brainerd et al. *Journal of Memory and Language*, *121*, 104286, (2021b). Finally, significant positive associations occurred between character-level variables and their corresponding word-level variables for both the first and second characters. The strength of these cross-level relationships varied across affective and lexico-semantic variables and may further be influenced by semantic transparency. Overall, our findings advance the understanding of affective and semantic features of Chinese characters and offer insights into the cross-level integration of characters’ and words’ lexical characteristics. The data reported in this paper are available at: https://osf.io/kh4yx.

## Introduction

The Chinese writing system is logographic and morpho-syllabic, characterized using individual characters that correspond to a single syllable and represent a unit of meaning (Hoosain, 1991). Each character is composed of multiple strokes, which sometimes form orthographic components known as radicals, functional units that often encode semantic or phonological information (Yin & Snowden Rohsenow, 1994). However, there is not always a direct link between a character’s orthography and its pronunciation at the syllabic level (e.g., only about 34–40% of characters share the same pronunciation as their phonetic radical, Law et al., 2009; Yum et al., 2014), making Chinese orthography distinct from alphabetic languages, such as Spanish or English, where there is a more transparent relationship between spelling and sound, allowing for phonological decoding.

Chinese characters frequently combine to form two-character words, which account for the majority of the Chinese lexicon. According to the Institute of Language Teaching and Research (1986), about 73.6% of Chinese words are composed of two characters (Myers, 2010; Packard, 2000). As not only words but also some characters carry meanings, there has been debates on whether words or characters should be considered the basic structural units in Chinese language (e.g., Wang, 2011). When two characters combine, the resulting word often represents a new concept that may not necessarily be inferable from the meanings of individual characters (Han et al., 2014; Chen et al., 2023a, b), such as 東西 (“thing” literally “east–west”). This bears resemblance to the formation of compound words in English, where the meaning of a compound word can range from compositional (or semantically transparent, e.g., *firewood*) to non-compositional (or semantically opaque, e.g., *blackmail*). Several studies have examined the role of constituents’ lexical characteristics in English compound word processing. For example, Gagné and Spalding (2016) examined the effect of semantic transparency on written production of compound words and found that the typing latency was sensitive to the morphemic structure, as well as semantic transparency of the first constituent (see also Kim et al., 2019, for related findings in lexical decision and naming). Investigating how orthographic, phonological, and semantic characteristics of Chinese characters and words interrelate provides valuable insights into the complexities of Chinese language processing and offers a comparative framework for cross-linguistic studies of compound word formation.

Understanding the lexical characteristics of Chinese characters and words is essential for several reasons. First, there is an increasing number of people worldwide learning Chinese as a second language (Tsung & Cruickshank, 2011; Gong et al., 2018), which highlights the practical significance of investigating its linguistic structure and function. Second, the distinctive features of the Chinese writing system, particularly its logographic nature, offer a unique perspective in psycholinguistics. This aspect of Chinese provides insights into cognitive processing that are critical for the development of universal theories of language processing, such as universal phonological hypothesis (Perfetti et al., 1992) and orthographic depth hypothesis (Katz & Frost, 1992). These hypotheses can be evaluated within the context of Chinese, which challenges the predominantly Anglocentric perspective in psycholinguistic research (Share, 2008).

Over the past decade, researchers have developed several Chinese word rating databases, which provide normed ratings for large pools of words across lexical variables, such as valence, arousal, concreteness, imageability, and familiarity (e.g., Chan & Tse, 2024; Ho et al., 2015; Su et al., 2022; Yee, 2017). Freely accessible to researchers, these normed ratings, which demonstrate high inter-rater reliability, become invaluable tools in the field, enabling experimental manipulations such as matching on specific lexical characteristics and facilitating investigations of the interrelationships among lexical variables (e.g., Chang & Lee, 2020; Wang et al., 2019). Despite the critical role of norming studies, the development of similar databases for Chinese characters; that is, basic orthographic unit of the language, has been largely limited to simplified script (Liu et al., 2007; Peng et al., 2024; Xue et al., 2024). This represents a significant gap, particularly for traditional Chinese script, which is still widely used in regions such as Hong Kong, Taiwan, and Macau. Traditional script differs from simplified script, which was introduced during writing reforms in the 1960 s to reduce stroke counts and facilitate literacy acquisition (e.g., 钟 vs. 鐘; simplified and traditional script for *clock*). The orthographic differences between simplified and traditional scripts may influence not only the ease of learning but also the interpretation and emotional connotations of characters (Chan & Tse, 2024). Moreover, existing character norming databases have revealed the intertwined relationships among affective and lexico-semantic variables in simplified scripts. For example, Pearson correlations among familiarity, concreteness, and imageability was found to be consistently positive, while these variables, together with valence were also negatively correlated with age of acquisition (Liu et al., 2007; Wang et al., 2019; Su et al., 2022; Peng et al., 2024). To fully understand the affective and semantic dimensions of Chinese characters, it is important to norm valence and arousal ratings for characters presented in traditional script, thereby addressing this gap in the literature.

The present study builds on the Chinese Lexicon Project (Tse et al., 2017, 2023), which compiled lexical decision and speeded naming performance data for 25,281 Chinese two-character words, along with various orthographic, phonological, and semantic variables. Our study has four research objectives:

- *Norming affective variables for Chinese characters*: We conducted a normed rating study for two affective variables, valence and arousal, on 3971 traditional Chinese characters, the base characters of the 25,281 words in Tse et al.’s (2017, 2023) database.
- *Examining inter-relationships among lexical variables of Chinese characters*: Using the newly normed valence and arousal ratings, as well as previously normed ratings of concreteness, imageability, and familiarity (Su et al., 2022), which were also provided by Chinese-speaking university students in Hong Kong, we investigated the inter-relationships among lexical characteristics at the character level.
- *Characterizing ambiguity measures*: We examined ambiguity measures, defined as the standard deviation of normed ratings across raters for each lexical variable, and their relationship with intensity measures (mean ratings) and explored quadratic relationships between intensity and ambiguity, as observed in previous work (Chan & Tse, 2024).
- *Cross-level analyses of character–word relationships*: We investigated the relationships between character-level and word-level lexical variables, including the moderating role of semantic transparency and the relative contributions of the first versus second character to word-level variables.

### Norming affective variables for Chinese characters and examining inter-relationships among lexical variables of Chinese characters

Research on the affective properties of Chinese characters remains relatively scarce, especially with respect to traditional Chinese characters compared to simplified ones. For example, Peng et al. (2024) normed valence and arousal ratings for 3827 simplified Chinese characters and found a weak positive correlation between character valence and familiarity (*r* =.125) and a weak negative correlation between character valence and age of acquisition (AoA; *r* = –.196). This suggests that characters with more positive valence tend to be more familiar and acquired earlier in life. Similarly, Xue et al. (2024) normed ratings for valence, emotional arousal, and other lexico-semantic variables (concreteness, imageability, and AoA) for 2500 simplified Chinese characters. They replicated Peng et al.’s findings of the negative valence–AoA relationship and also found positive correlations between emotional arousal and imageability and between valence and imageability. However, Xue et al. reported that the relationships of valence and arousal, arousal and AoA, valence and concreteness, and arousal and concreteness were varied depending on the lexical composition and positioning of these characters within their corresponding two-character words. The authors did not provide a detailed explanation for these unsystematic patterns, suggesting a need for further investigation into how affective variables are associated with lexico-semantic variables within a larger pool of Chinese characters in another (traditional) script.

Beyond the relationships between affective and lexico-semantic variables, some studies have considered the associations among lexico-semantic variables of Chinese characters. For example, the correlation between AoA and other lexical variables, such as familiarity, concreteness, and imageability, has been consistently negative in previous studies (Liu et al., 2007; Wang et al., 2019; Su et al., 2022). These findings suggest that characters acquired earlier in life tend to be more familiar, more concrete, and easier to form mental images. Also, robust positive correlations were reported among imageability, concreteness, and familiarity, such as strong imageability–concreteness correlations (*r =.*796 in Liu et al., 2007; *r =.*804 in Wang et al., 2019; *r =.*923 in Su et al., 2022), moderate imageability–familiarity correlations (*r =.*641 in Liu et al., 2007; *r =.*488 in Su et al., 2022), and moderate concreteness–familiarity correlations (*r =.*470 in Liu et al., 2007; *r =.*467 in Su et al., 2022). These suggest that characters perceived as more familiar are also more likely to be seen as concrete and easily imaginable, highlighting the intertwined nature of these lexico-semantic variables. This suggests the importance of taking related variables into account when researchers examine the relationships among lexico-semantic variables of Chinese characters and tease apart their unique impact on word recognition performance.

Other than examining relationships among lexical variables within Chinese characters, Xue et al. (2024) compared the inter-relationships of lexico-semantic variables within two-character words with those within their characters and observed both similarities (e.g., concreteness–imageability correlation) and differences (e.g., AoA–imageability correlation) between character and word levels. They argued that the words and their corresponding characters are inherently associated but also independent to some extent. However, it is not clear why some variable relationships remain consistent between characters and words while others diverge. Given that Xue et al.’s study involved only about 7311 two-character words and 2500 characters, it is important to replicate and extend these findings with a larger pool of two-character words and characters.

### Valence ambiguity and intensity–ambiguity relationship

An emerging area in psycholinguistic and memory research concerns attribute ambiguity, which refers to variability in individuals’ subjective ratings of lexical attributes. Valence ambiguity, for instance, arises because personal experiences shape differing emotional reactions toward the same word. For example, the word “dog” may evoke positive feelings in some individuals, yet elicit negative evaluations from others who have experienced adverse encounters (e.g., being bitten). Despite its relevance, valence ambiguity, defined as the standard deviation of valence ratings across raters, was largely overlooked until recent investigations by Brainerd and his colleagues (Brainerd et al., 2021a, b; Chang & Brainerd, 2023). Brainerd (2018) reported that greater valence ambiguity weakens the typically robust relationship between valence and arousal. Similarly, increased ambiguity in arousal ratings also attenuates the valence–arousal association (Brainerd, 2018; Brainerd et al., 2021a, b; Mattek et al., 2017; Chang & Brainerd, 2023).

Expanding beyond valence and arousal, Brainerd and his colleagues (2021) identified a quadratic relationship between intensity (mean ratings) and ambiguity (standard deviation of ratings) for several other lexico-semantic variables, including concreteness, familiarity, and imageability. Specifically, ambiguity tends to peak at moderate intensity levels, forming an inverted-U-shaped pattern where subjective ratings at intermediate intensities are associated with maximal variability (Brainerd et al., 2021a, b). Chan and Tse (2024) replicated most of these relationships using a large dataset of 25,281 Chinese two-character words from Tse et al.’s Chinese Lexicon Project (2017, 2023). Their findings confirmed the quadratic intensity–ambiguity relationship for valence, arousal, concreteness, and imageability, while not for familiarity. Moreover, they demonstrated that ambiguity in valence and arousal moderates the asymmetrical U-shaped relationship between these two attributes, suggesting distinct processes for intensity and ambiguity during lexical processing.

To date, however, no research has tested whether these intensity–ambiguity patterns generalize to Chinese characters, the more fundamental units of the Chinese writing system. As individual characters are the building blocks of two-character words, examining intensity–ambiguity relationships at the character level is critical for determining the consistency of these patterns across different lexical levels. Following Brainerd and his colleagues (2021), we quantify the ambiguity of a lexical variable as the standard deviation of its ratings across raters and intensity as the corresponding mean rating across raters.

### Cross-level analyses of character–word relationships

Examining the relationship between the lexical variables of two-character words (e.g., valence, arousal, imageability, concreteness, and familiarity) and those of their constituent characters is a critical step in understanding Chinese word processing. For example, do words composed of two characters with more negative valence exhibit overall negative valence as a whole? The potential consistency of lexico-semantic and affective variables between two-character words and their corresponding characters has important implications for theoretical models of Chinese word processing. Given the linguistic structure of Chinese words; that is, combinations of constituent characters that typically, though not always, carry individual meanings, theoretical models often emphasize the interplay between character-level and word-level representations. Some models propose that characters and multi-character words are represented separately (e.g., Li et al., 2016; Taft et al., 1999; Tan & Perfetti, 1999), whereas other models explicate the connections between the individual characters and the word (e.g., Li et al., 2016; Peng et al., 1999; Zhou & Marslen-Wilson, 2000) (see Tse & Yap, 2018; Tse et al., 2024, for their detailed introduction). These suggest that those of its characters may partially shape the lexical characteristics of a two-character word.

The hierarchical organization of the Chinese lexicon implies that word processing involves both bottom-up and top-down processes. Specifically, early stages of processing may involve the extraction of character-level representations, which are then integrated to form word-level representations. For example, a two-character word may activate semantic nodes corresponding to each character, leading to overlapping activations in affective (e.g., valence, arousal) and perceptual (e.g., imageability, concreteness) dimensions. From this distributed and interactive perspective, the global semantic profile of a two-character word could reflect, to some extent, the sum or integration of the lexical attributes of its characters.

While word-level variables are generally expected to correlate with character-level variables, two additional issues warrant consideration: the role of semantic transparency and the relative contributions of the first and second characters to the word-level lexical variables.

Semantic transparency refers to the degree of semantic relatedness between a word and its two characters (Tse et al., 2017). It has long been recognized as a key moderator of how character-level information contributes to word-level representations in Chinese (e.g., Myers et al., 2004; Tse & Yap, 2018; Tse et al., 2024). In highly transparent words, each character’s meaning aligns closely with the meaning of the whole word, such that the activated lexico-semantic and affective information of the characters (e.g., valence and concreteness) is more coherent with that of the word. By contrast, in opaque words, the relationship between the word and its characters is obscure or can be idiomatic in meaning; that is, the meanings of the constituent characters may conflict with the meaning of the whole word. Hence, the relationship between the characters’ lexical variables and the word’s lexical variable is expected to be stronger for transparent words (e.g., 花園 “garden” literally “flower-park”) than opaque words (e.g., 沙發 “sofa” literally “sand-deliver”).

The relative contributions of the first and second characters to the lexical variables of the whole word also warrant further investigation. Using a megastudy approach with over 17,000 two-character words, Tse et al. (2024) examined how both character-level and word-level lexical variables influence lexical decision and naming performance. They found that, in lexical decision tasks, the effects of lexical variables were comparable regardless of whether the variable was associated with the first or second character. In contrast, naming tasks revealed stronger influences from lexical variables linked to the first character. These results suggest that readers tend to process both characters in parallel when making lexical decisions but rely on a sequential processing strategy when reading words aloud.

This raises several questions: when evaluating lexico-semantic and affective characteristics, are word-level lexical variables inherently more influenced by the attributes of the first characters than those of the second characters? If so, does the degree of this influence vary across different lexico-semantic and affective variables? For example, might word valence be more strongly associated with the valence of the first characters compared to that of the second characters? In contrast, word arousal might rely on a more balanced contribution from both characters.

To our understanding, current models of Chinese word processing do not explicitly predict whether the first or second character would have a stronger association with word-level variables. Addressing these questions requires systematically comparing the associations between word-level variables and the lexical variables of the first and second characters across multiple lexical variables. Despite the substantial theoretical implications, to our knowledge, no study has systematically examined the correlation between character-level and word-level lexical variables across a wide range of dimensions. Existing research, such as Tse et al. (2023) and Chan and Tse (2024), has primarily focused on word-level variables, leaving a significant gap in understanding the intertwining relationship between character-level and word-level lexical variables. With the availability of newly normed valence and arousal ratings for single characters (this study) and previously normed lexico-semantic variables (e.g., concreteness, imageability, familiarity; Su et al., 2022), as well as the well-compiled lexical variables of two-character words in Tse et al. (2023), it is now possible to conduct systematic cross-level analyses.

### The present study

The present study aims to investigate the relationships among affective and lexico-semantic variables in Chinese characters presented in traditional script, building on 25,281 two-character words and their corresponding 3971 characters compiled in the Chinese Lexicon Project (Tse et al., 2017, 2023). We also compare these relationships within this character pool to see if it replicates the findings previously observed in the word pool (Chan & Tse, 2024). Moreover, we examine the intensity–ambiguity relationships in Chinese characters and compare these findings with those previously reported for two-character words (Chan & Tse, 2024). This analysis explores whether quadratic relationships between intensity and ambiguity, observed in variables such as valence, arousal, concreteness, and imageability, extend to the character level. Furthermore, we conduct cross-level analyses to examine the relationships between character-level variables and word-level variables, how they can be moderated by semantic transparency, and how the lexical variables of the first and second characters are associated differentially with the lexical variables of the whole word. Taken together, these findings aim to advance our understanding of the affective and lexico-semantic dimensions of Chinese characters and their reflection in word features.

## Method

### Participants

Two hundred and fifteen native Cantonese-speaking students from the Chinese University of Hong Kong (CUHK), the same population as in Tse et al. (2017), were recruited to participate in online rating tasks for valence and arousal, conducted in two separate sessions. Participants who did not complete the experiment (*N* = 38), responded too quickly (i.e., more than 50% of responses were < 200 ms; *N* = 9), encountered network server issues (*N =* 4), completed the tasks more than once (*N =*3), or misunderstood the instructions (*N =* 1) were excluded. This process left data from 160 participants for the final analyses. Participants ranged in age from 18 to 24 years (*M* = 19.96, *SD* = 1.37) and were predominantly female (68.75%). This study was approved by the Survey and Behavioral Research Ethics Committee at the Chinese University of Hong Kong (SBRE-23-0238) and was conducted in accordance with relevant guidelines and in accordance with the Declaration of Helsinki. Prior to their participation in the study, all participants provided informed consent. All participants received monetary compensation of HK$100 upon completing both sessions.

### Materials and procedure

The 3971 characters were base characters obtained from Tse et al.’s (2017) pool of 25,281 two-character words. These characters were randomly divided into eight lists (496–497 characters each). Each list was assigned to 20 participants for rating. Valence and arousal were assessed using Bradley and Lang’s (1994) nine-point Self-Assessment Manikin scale (1 = *extremely negative/calm*; 9 = *extremely positive/excited*). Familiarity, imageability, concreteness, age of acquisition (AoA), and semantic radical transparency ratings were obtained from Su et al. (2022), where 4376 characters were rated by 20 participants each. The semantic radical transparency ratings were collected for 3126 phonetic characters, with those that had undergone orthographic changes being removed. Semantic transparency was rated by 20 participants per word on a seven-point scale for their semantic relatedness between word and corresponding characters, where 7 = *most related/transparent* (see Tse et al., 2017, for more details). Word-level variables, including valence, arousal, familiarity, concreteness and imageability, were retrieved from Chan and Tse (2024), which involved ratings for 25,281 two-character words by 20 participants per word.

Data collection was conducted online using PsychoPy (Peirce et al., 2019) hosted on Pavlovia.org. Participants were provided with the URL link via e-mail. Each participant completed two separate rating sessions: one for valence and one for arousal. To minimize task fatigue and ensure independent evaluations, each participant rated one of the eight character lists for valence and a different list for arousal. The order of the two sessions was counterbalanced across participants. After completing the first session, participants received the URL link for the second session via e-mail. The mean time taken for each session was about 35.52 min (*SD* = 165.58). The mean time lag between two sessions was about 2.04 days (*SD* = 1.51).

During each rating session, characters were presented one at a time on the screen and remained on the screen until a key response was recorded. Participants were instructed to rate their first impression of each character as quickly as possible and provided with definitions and examples of valence and arousal before the task to ensure their clear understanding of the rating criteria. Similar to the typical procedure in other norming studies (e.g., Chan & Tse, 2024), no attention check was implemented, allowing the present findings to be directly compared with those of previous studies.

## Results

The mean valence and arousal ratings for all 3971 characters can be accessed here: https://osf.io/kh4yx. Descriptive statistics of valence, arousal, familiarity, imageability, concreteness, age of acquisition (AoA), and their ambiguities (i.e., standard deviations) are summarized in Table 1. All mean values for these variables were based on ratings from 20 participants per character.

**Table 1:** Descriptive statistics of characters’ lexical variables (*N* = 3971)

Variables	Mean	*SD*	Range	Skewness	Kurtosis
Valence (9-point scale)	4.782	.958	1.75–7.95	–.185	2.92
Arousal (9-point scale)	3.930	.780	2.10–7.20	.672	3.35
Familiarity (7-point scale)	6.079	.669	2.75–6.95	–.126	4.41
Concreteness (7–point scale)	3.790	1.197	1.15–6.75	.220	2.29
Imageability (7-point scale)	3.924	1.158	1.23–6.82	.0581	2.28
Age of acquisition (7-point scale)	4.235	1.018	1.10–6.71	–.221	2.67
Valence ambiguity	1.58	.331	.37–.2.68	–.0874	3.08
Arousal ambiguity	2.24	.307	1.14–3.19	–.199	2.92
Familiarity ambiguity	1.17	.431	.22–2.39	.106	2.08
Concreteness ambiguity	1.68	.309	.41–2.52	–.615	3.63
Imageability ambiguity	1.81	.303	.56–2.69	–.640	3.67
Age of acquisition ambiguity	1.17	.230	.44–2.32	.371	3.71

*Note*. Familiarity, concreteness, imageability, and age of acquisition ratings are retrieved directly from Su et al. (2022; *N =*3604)

**Fig. 1:**
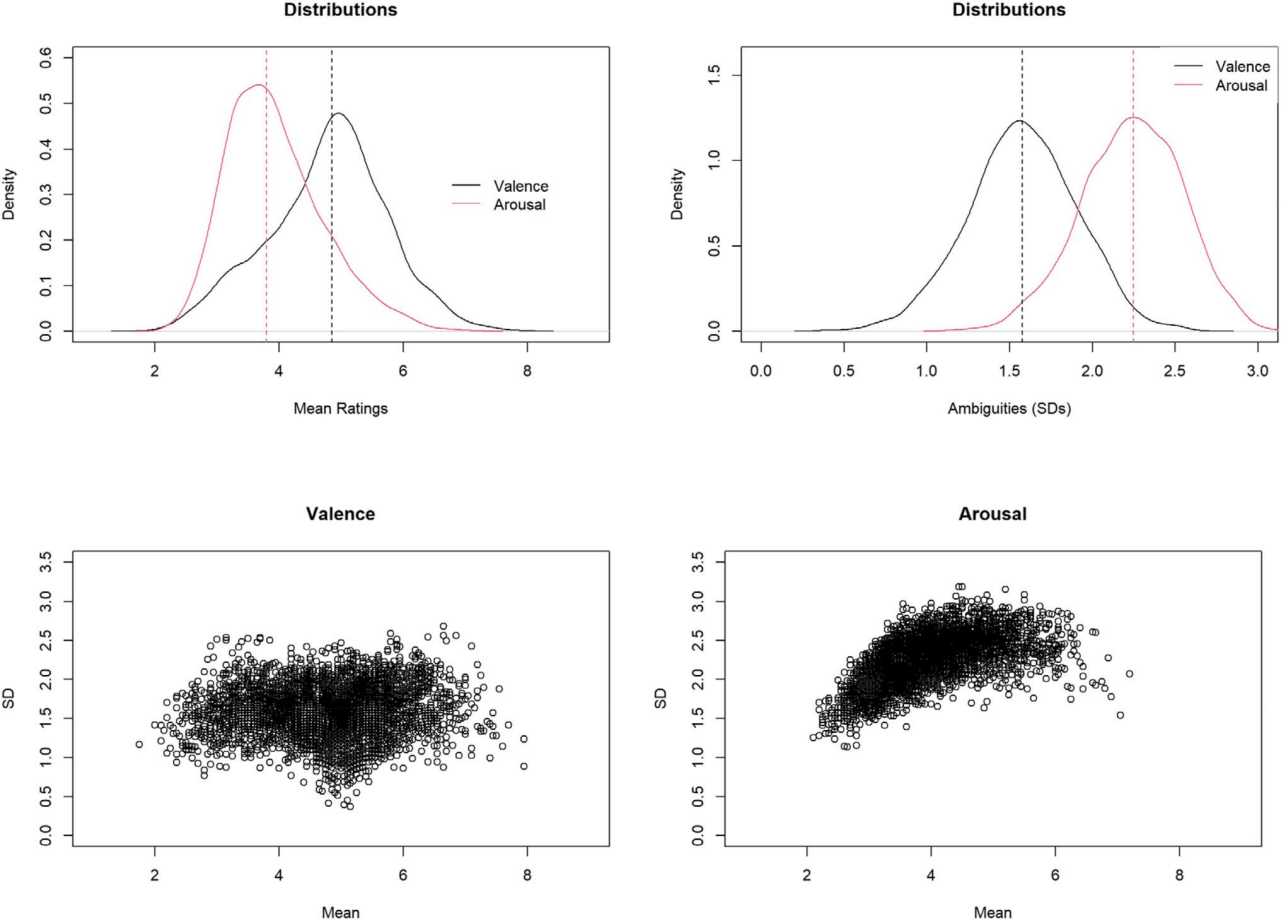
Distribution of mean and ambiguities of valence and arousal. *Note*. Top left: Distributions of valence and arousal ratings. Top right: Distributions of valence and arousal ambiguities. Bottom left: Scatterplots for the variability of valence. Bottom right: Scatterplot for the variability of arousal. Dashed lines indicate medians

The distributions of valence, arousal, and their variabilities are shown in Fig. 1. Valence was normally distributed, with 54.32% characters rated above the mean. Variability in valence ratings was smaller for characters with mid-range valence scores. Arousal, on the other hand, exhibited a positive skew, with only 44.60% of characters rated above the mean. Variability in arousal ratings was lower for characters rated as less arousing. Valence ambiguity (standard deviation of valence ratings) was normally distributed, with 49.46% of characters’ valence ambiguity above the mean. Similarly, arousal ambiguity (standard deviation of arousal ratings) followed a normal distribution, with 50.82% characters’ arousal ambiguity above the mean.

### Reliability

Inter-rater reliability for valence and arousal ratings was assessed using split-half correlations, corrected by the Spearman–Brown formula. Participants were divided into two equal groups based on whether their numbers were odd or even. Corrected correlations for valence ratings across the eight lists ranged from.78 to.94 (.78,.92,.94,.84,.86,.91,.92, and.88), while for arousal ratings, they ranged from.61 to.84 (.79,.65,.68,.61,.78,.81,.84, and.78). These results suggest that valence ratings were generally more reliable than arousal ratings, consistent with findings in previous studies (Warriner et al., 2013; Chan & Tse, 2024).

### Correlation with previous studies

Since no prior studies have normed affective ratings of traditional Chinese characters, we compared our results with simplified Chinese character norming studies (Peng et al., 2024, *N =* 3827; Xue et al., 2024, *N =* 2500). Valence ratings in our study were strongly correlated with those reported in Peng et al. (.82) and Xue et al. (.79). Arousal ratings also showed positive but moderate correlations with these studies (.55 in Peng et al. and.51 in Xue et al.). The weaker correlation for arousal may reflect differences in the populations sampled (mainland China in prior studies vs. Hong Kong in the present study), as arousal ratings are susceptible to cultural and individual differences (Montefinese et al., 2014).

### Relationships among lexical variables

**Fig. 2:**
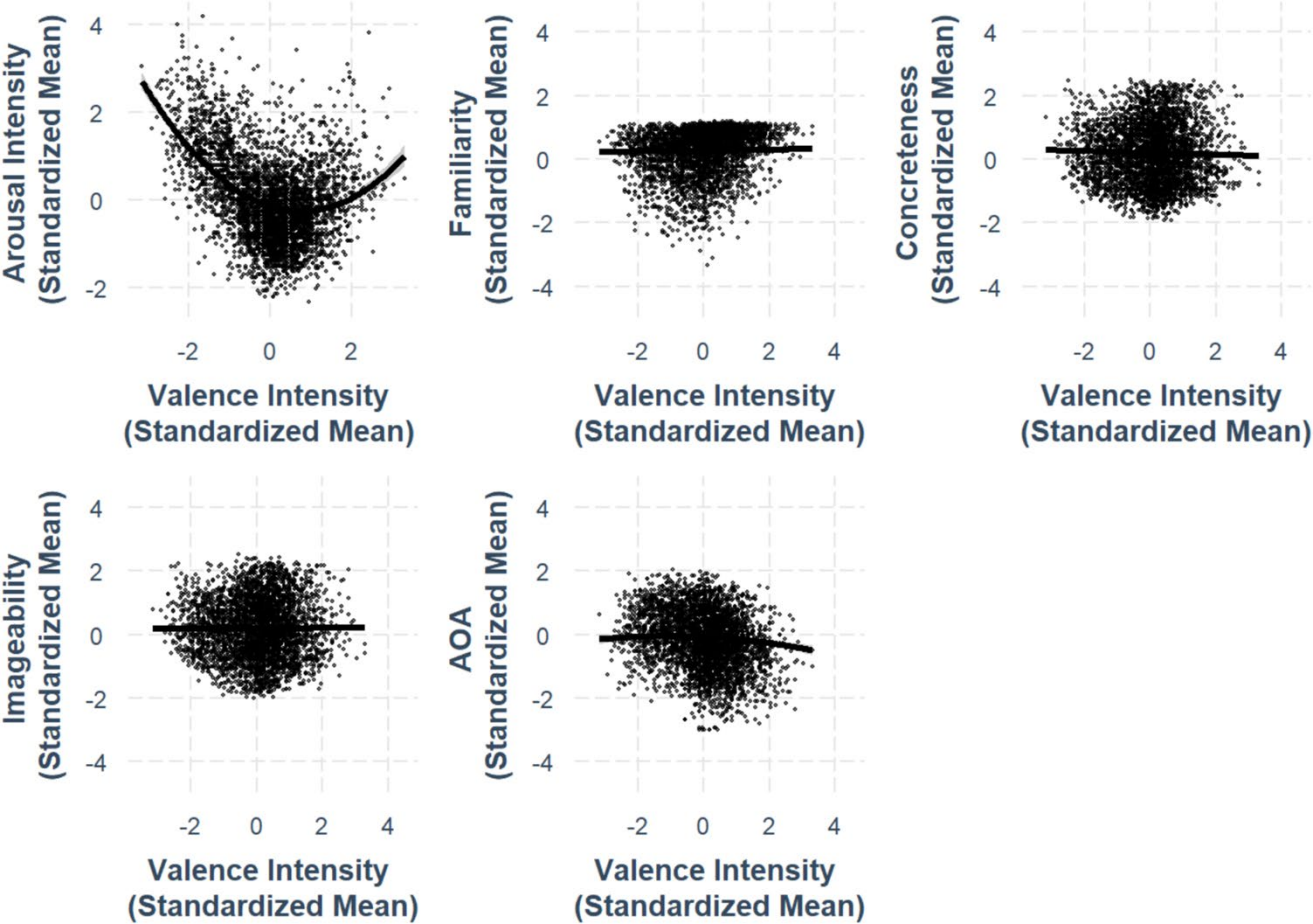
Relationships between valence and other lexico-semantic variables

**Fig. 3:**
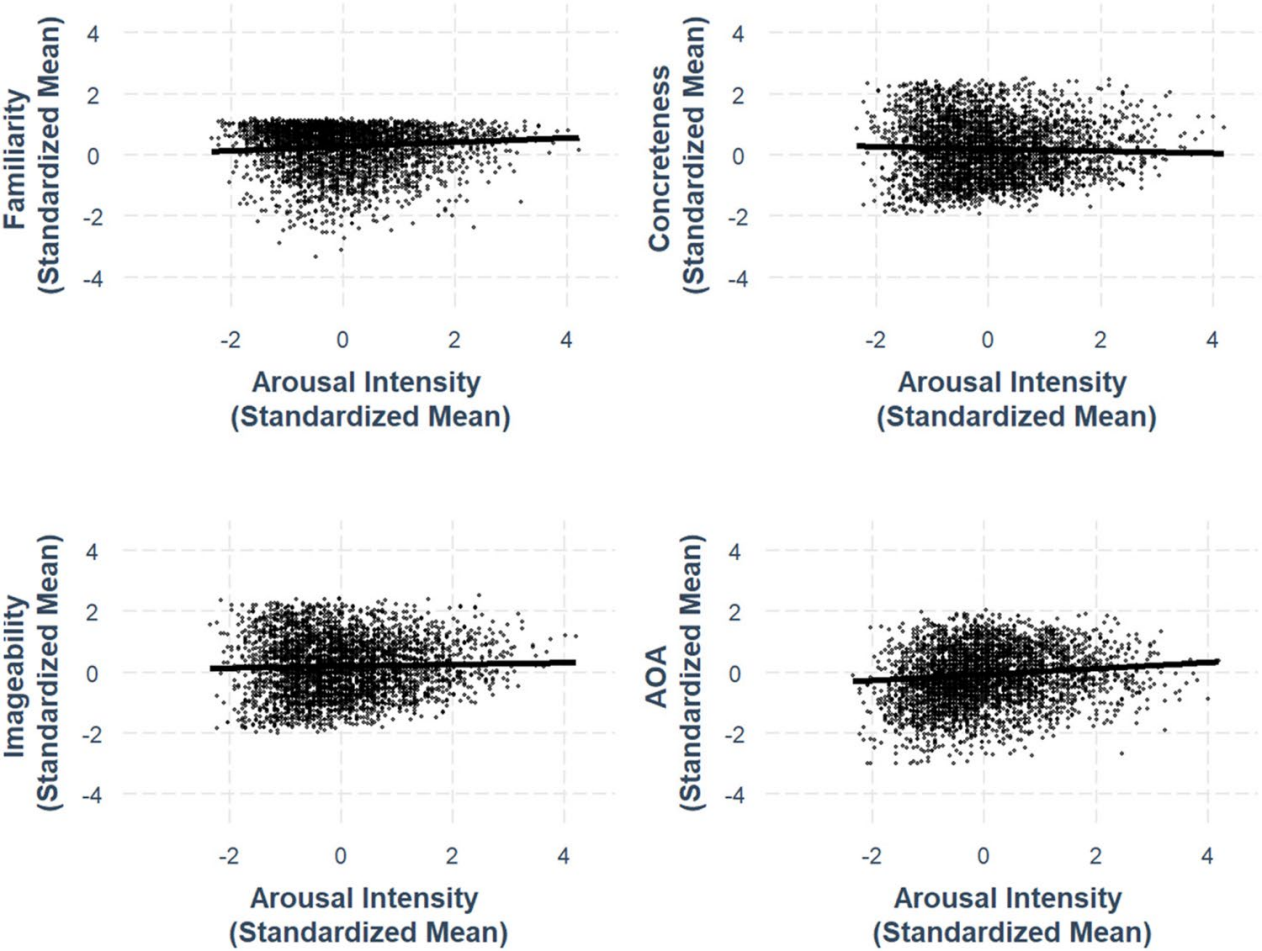
Relationships between arousal and other lexico-semantic variables

**Fig. 4:**
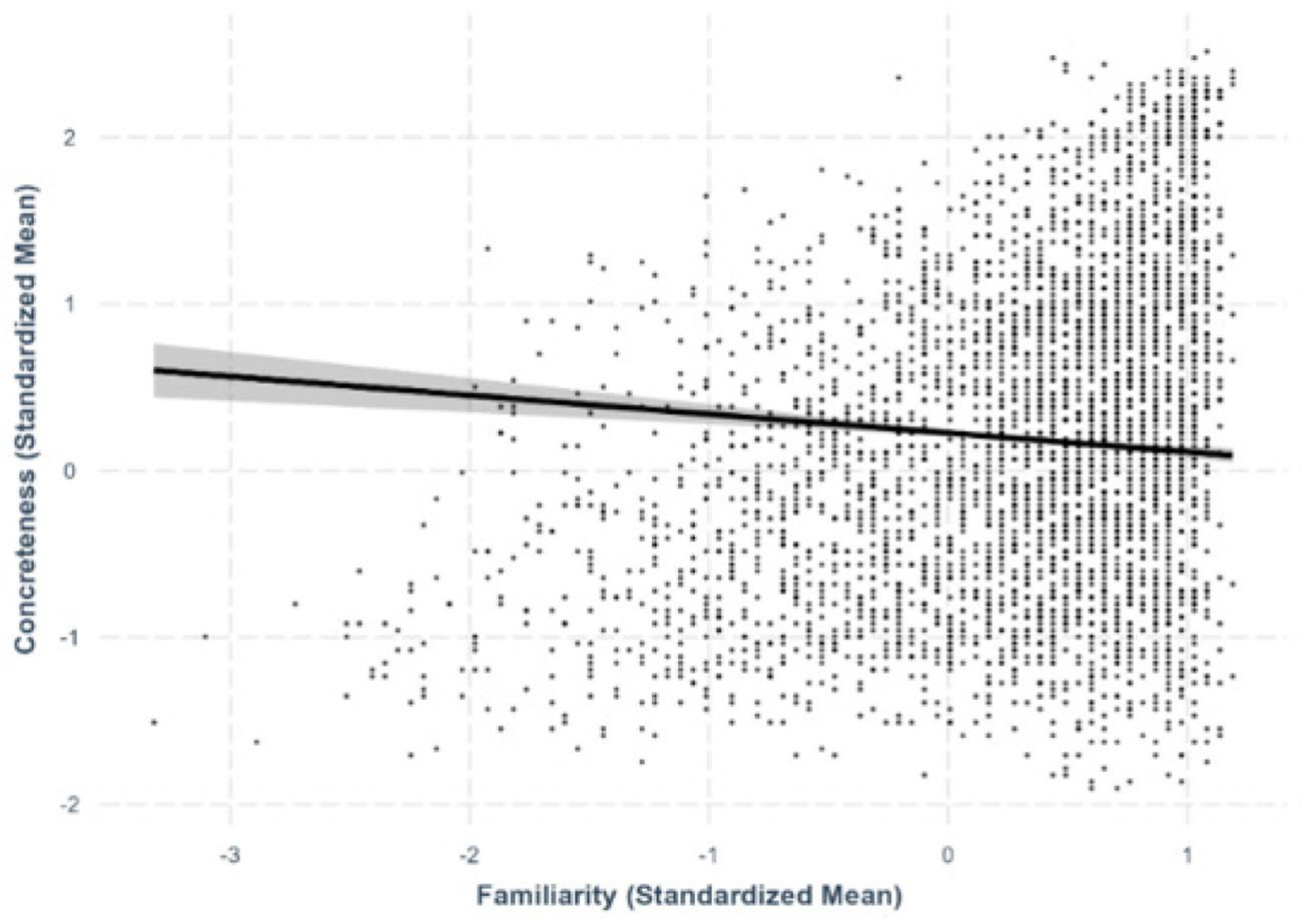
Familiarity–concreteness relationship

**Fig. 5:**
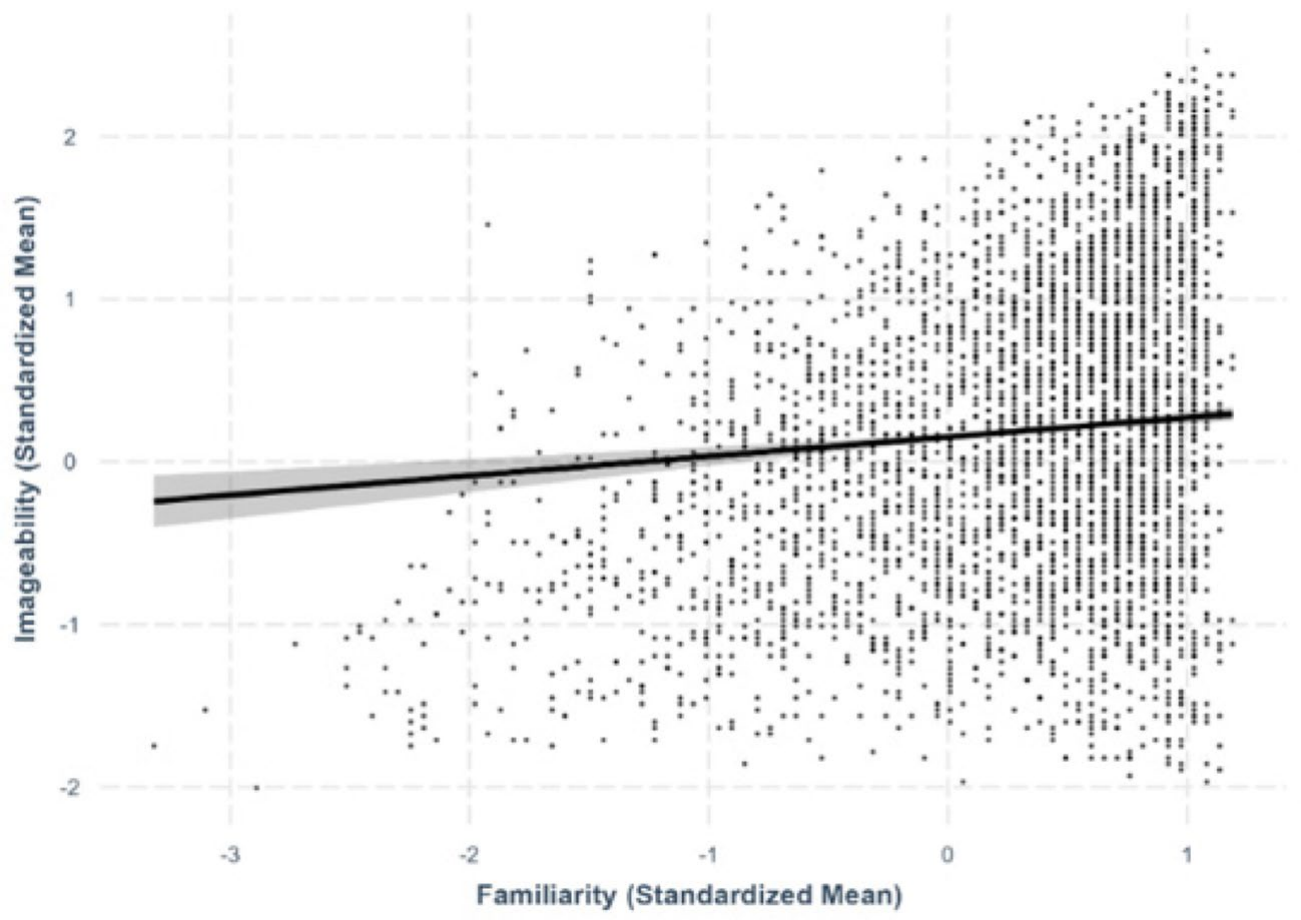
Familiarity–imageability relationship

**Fig. 6:**
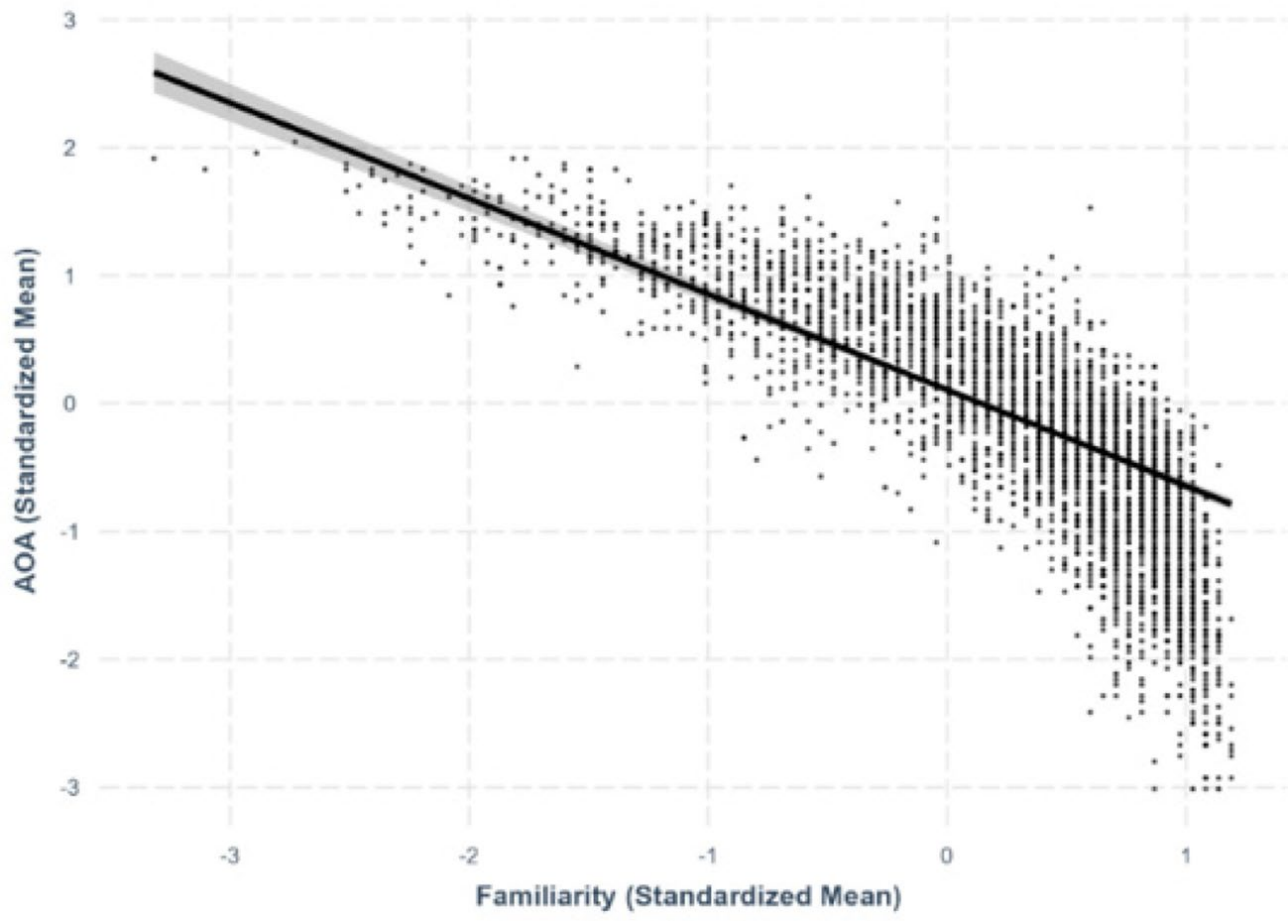
Familiarity–age-of-acquisition relationship

**Fig. 7:**
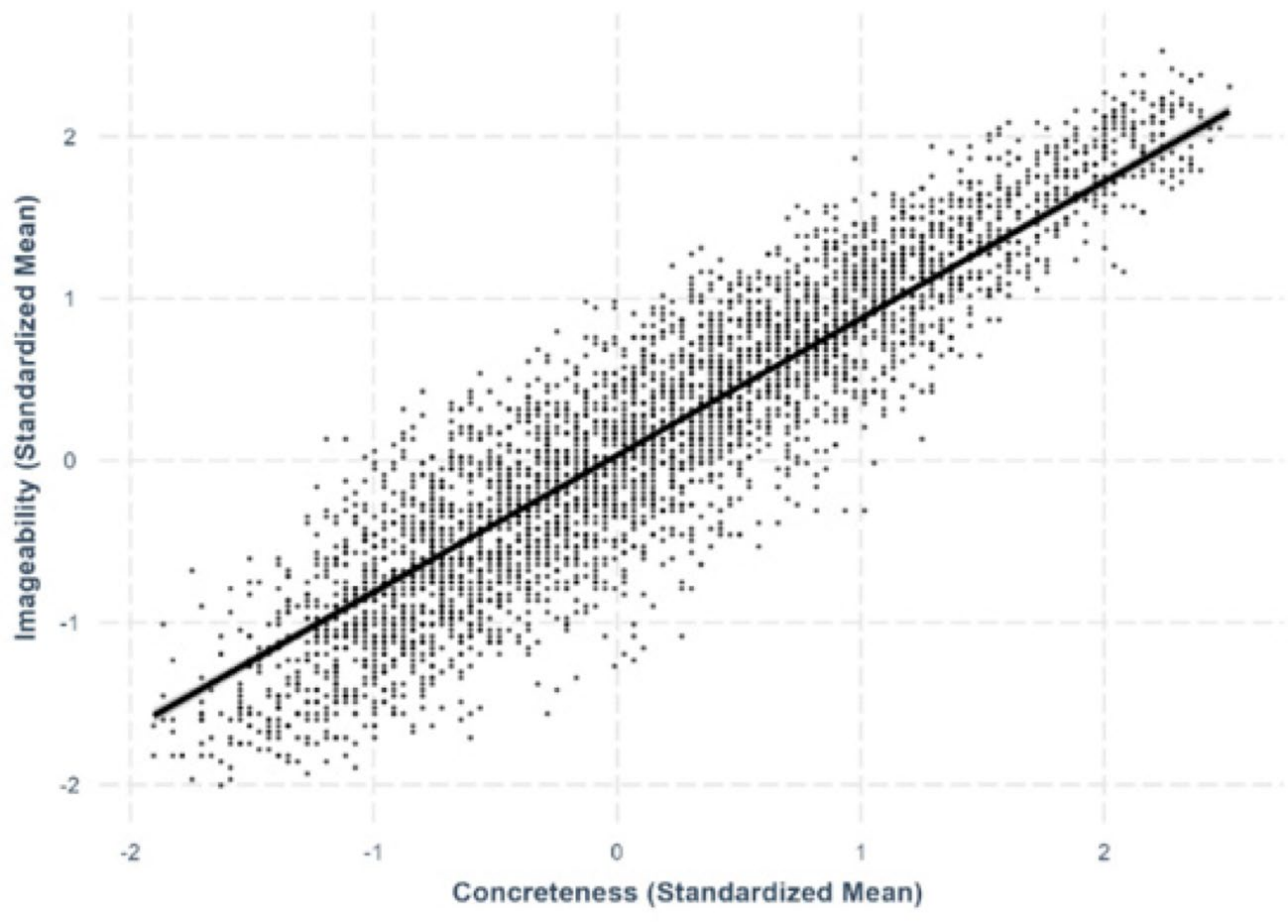
Concreteness–imageability relationship

**Fig. 8:**
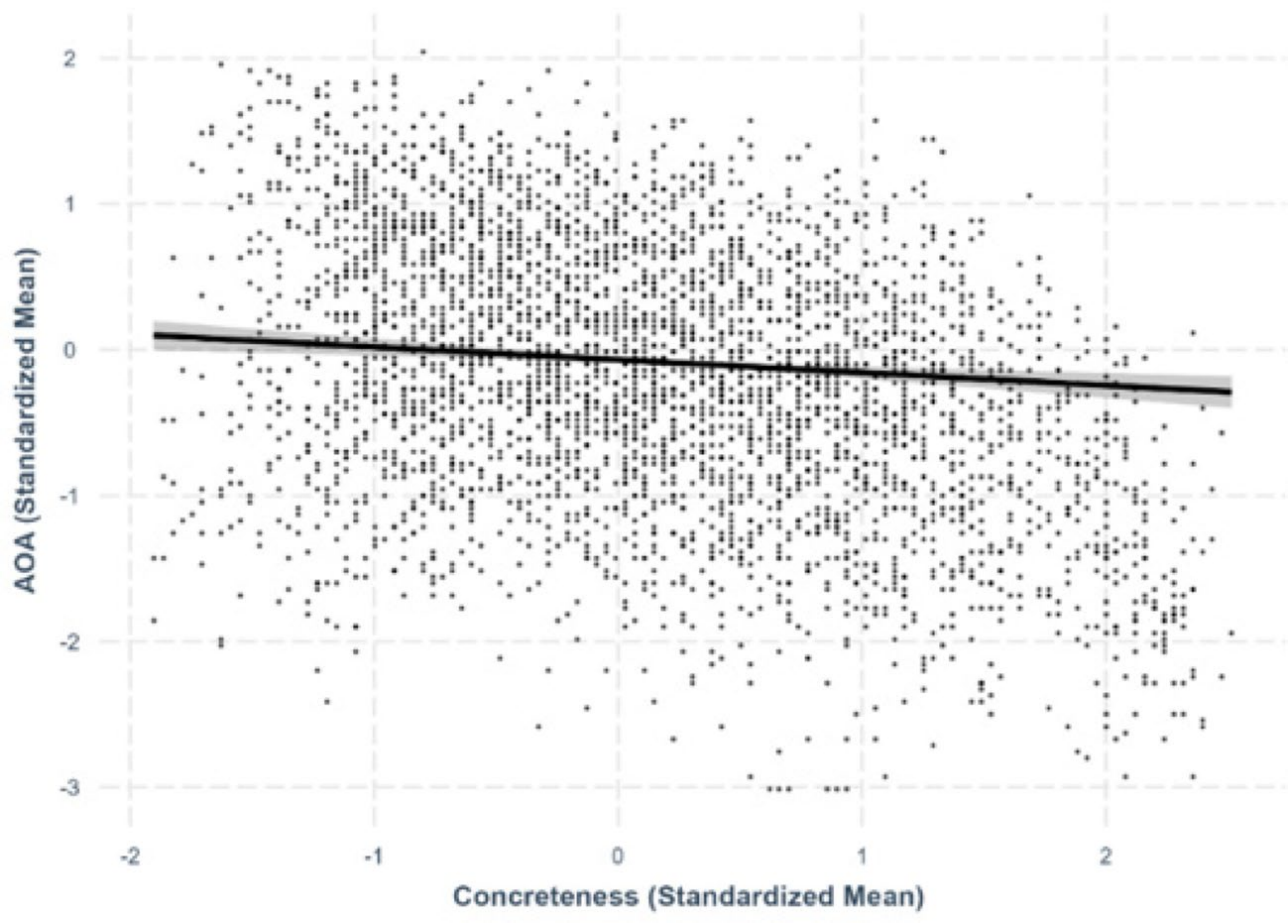
Concreteness–age-of-acquisition relationship

**Fig. 9:**
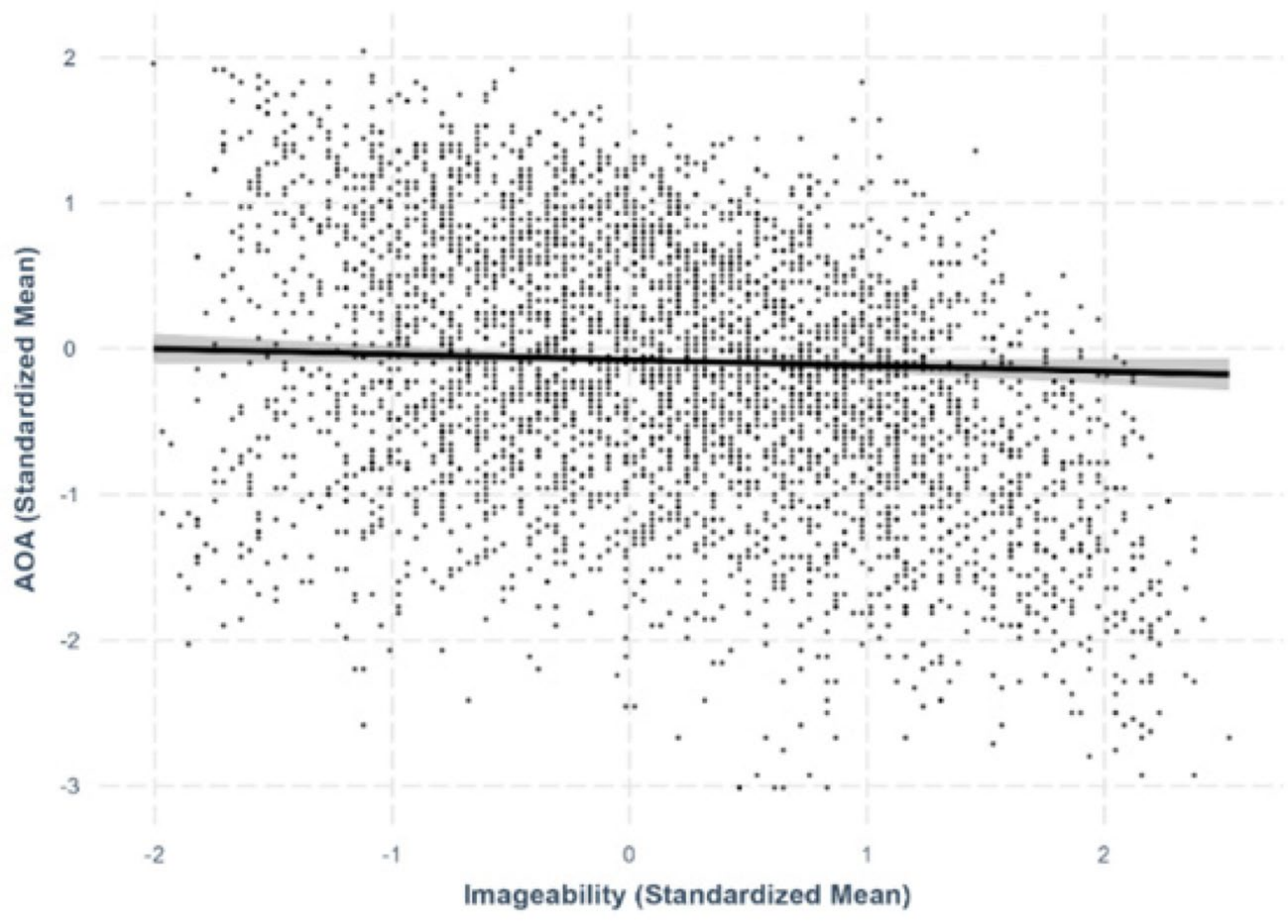
Imageability–age-of-acquisition relationship

**Fig. 10:**
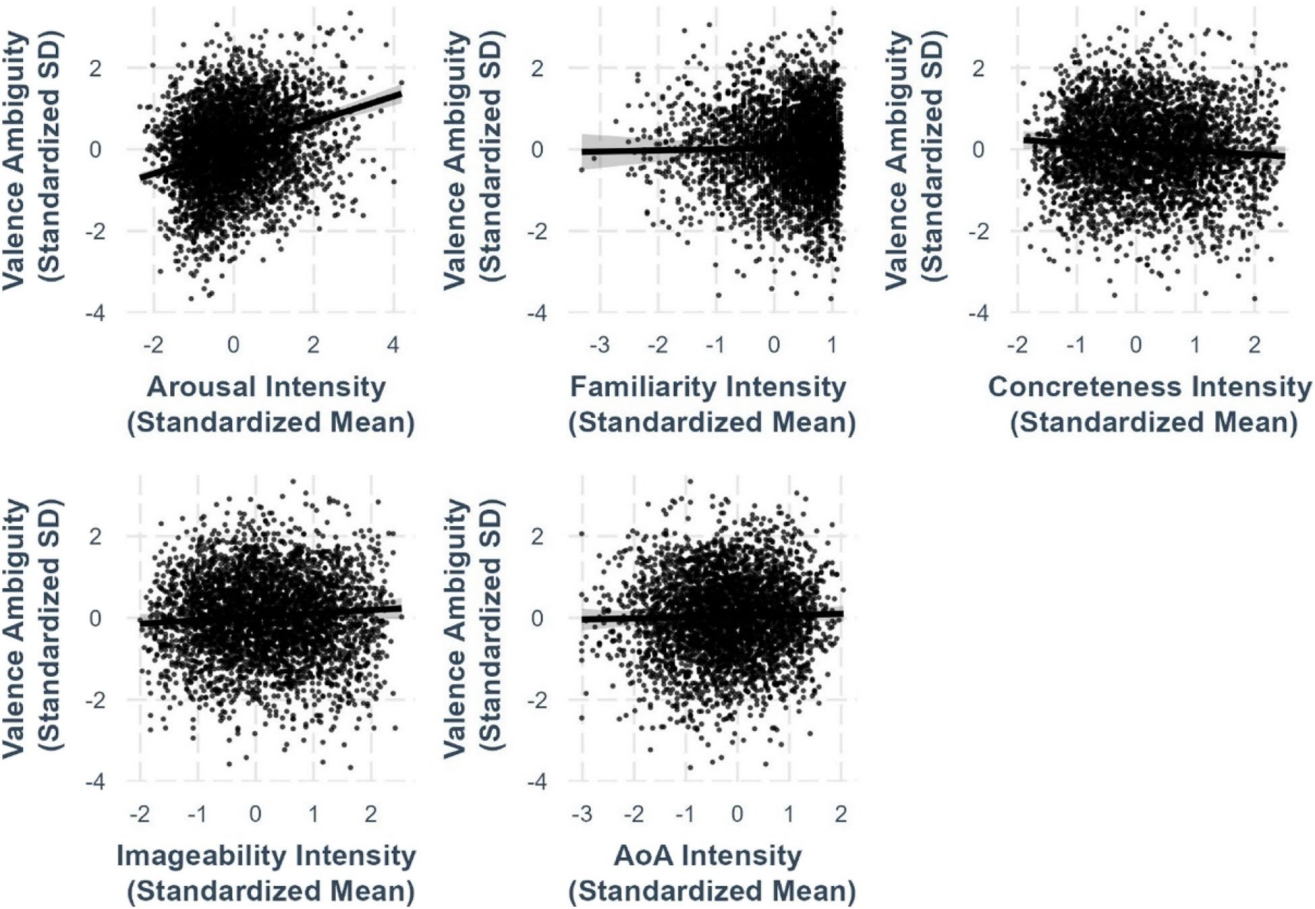
Intensity–valence ambiguity relationships

**Fig. 11:**
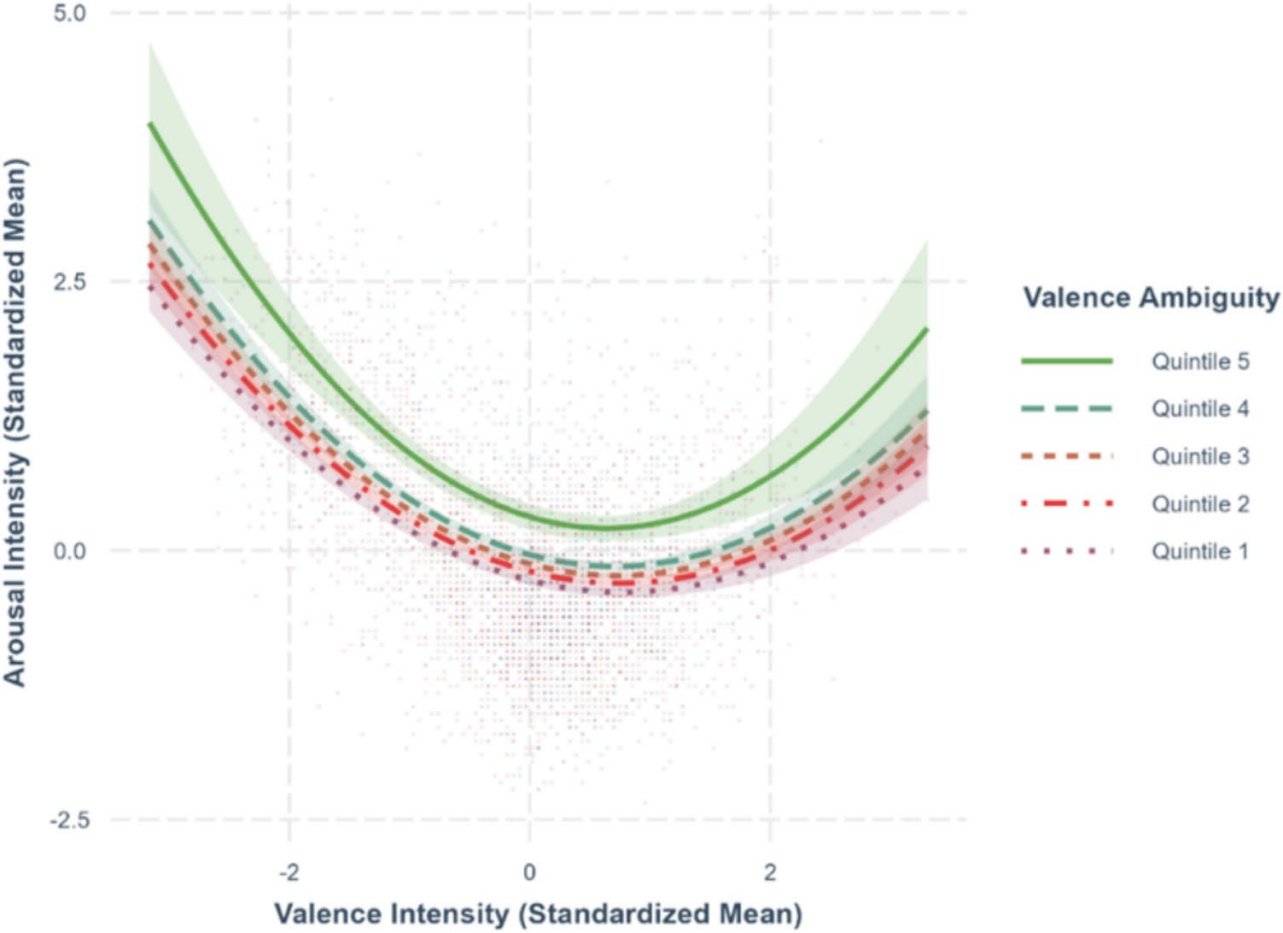
Valence–arousal relationship moderated by valence ambiguity

**Fig. 12:**
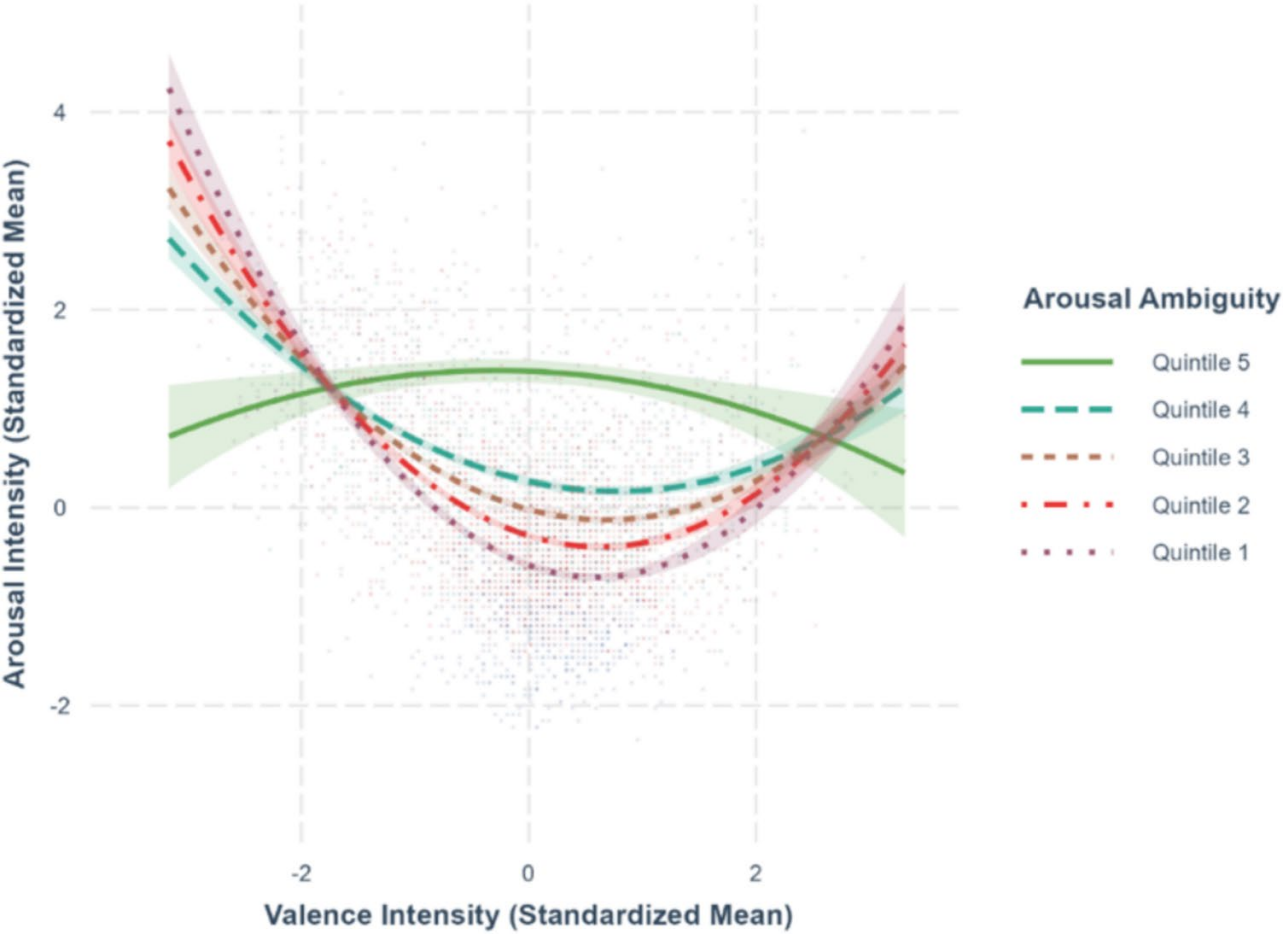
Valence–arousal relationship moderated by arousal ambiguity

**Fig. 13:**
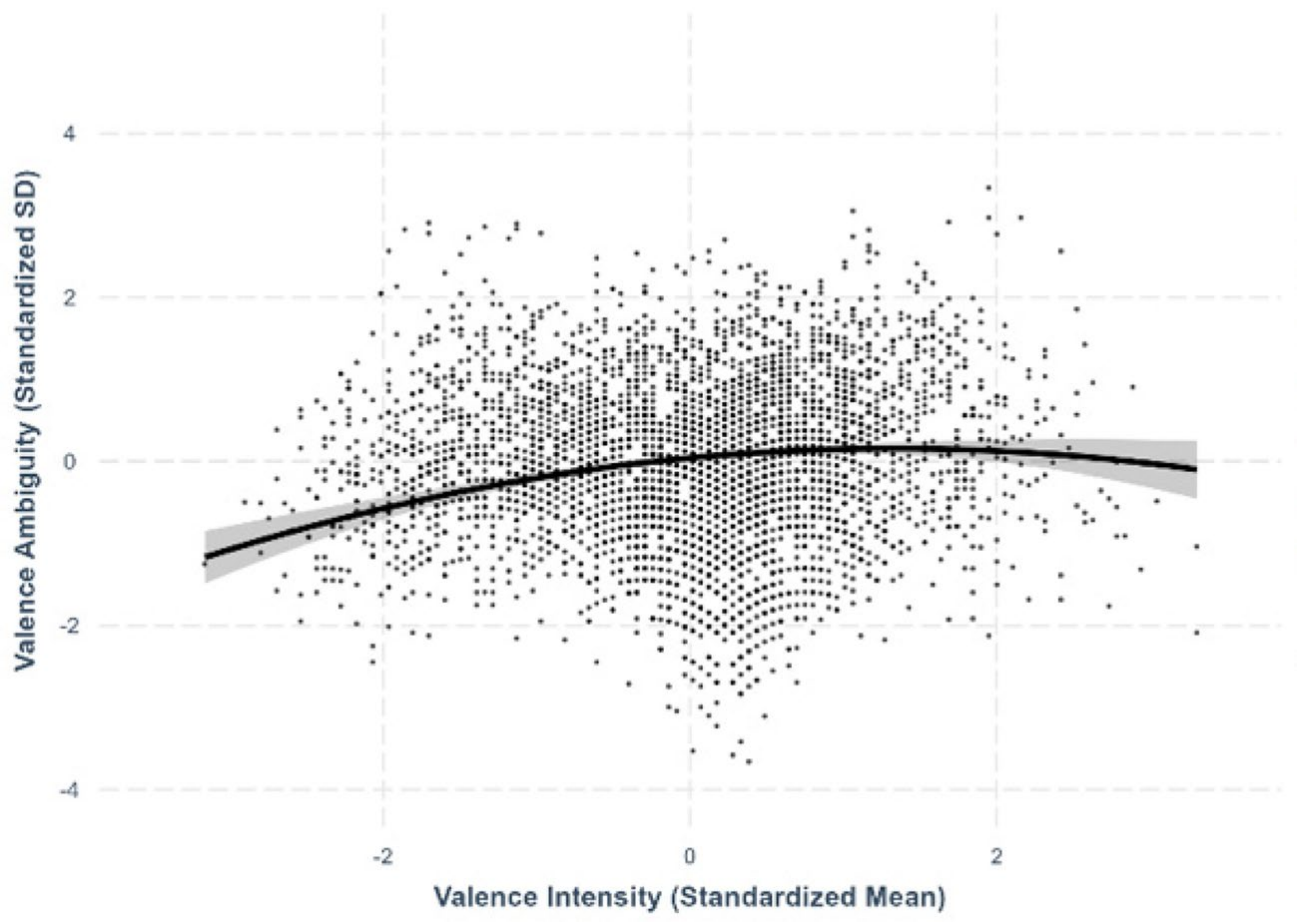
Valence–valence ambiguity

**Fig. 14:**
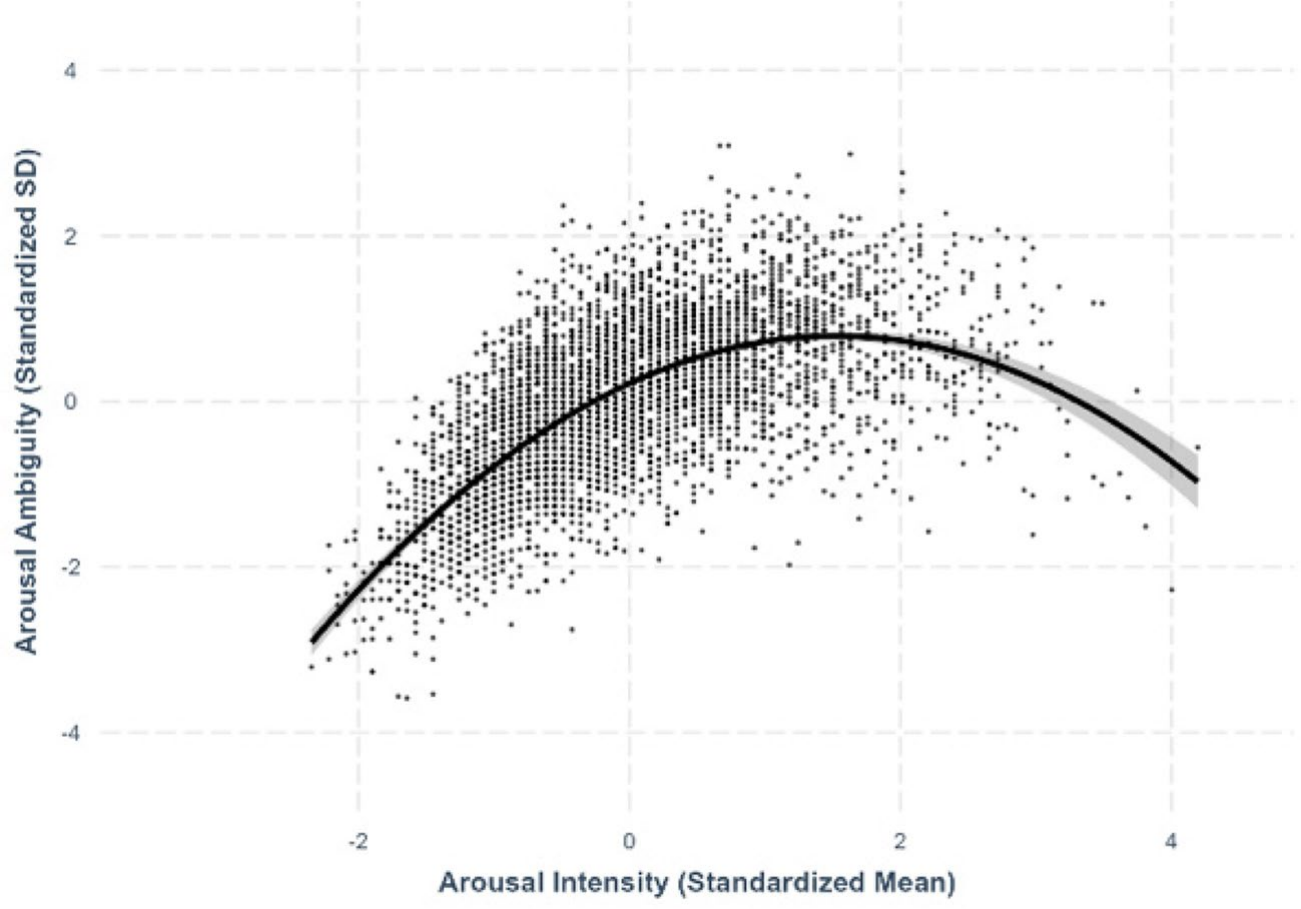
Arousal–arousal ambiguity

**Fig. 15:**
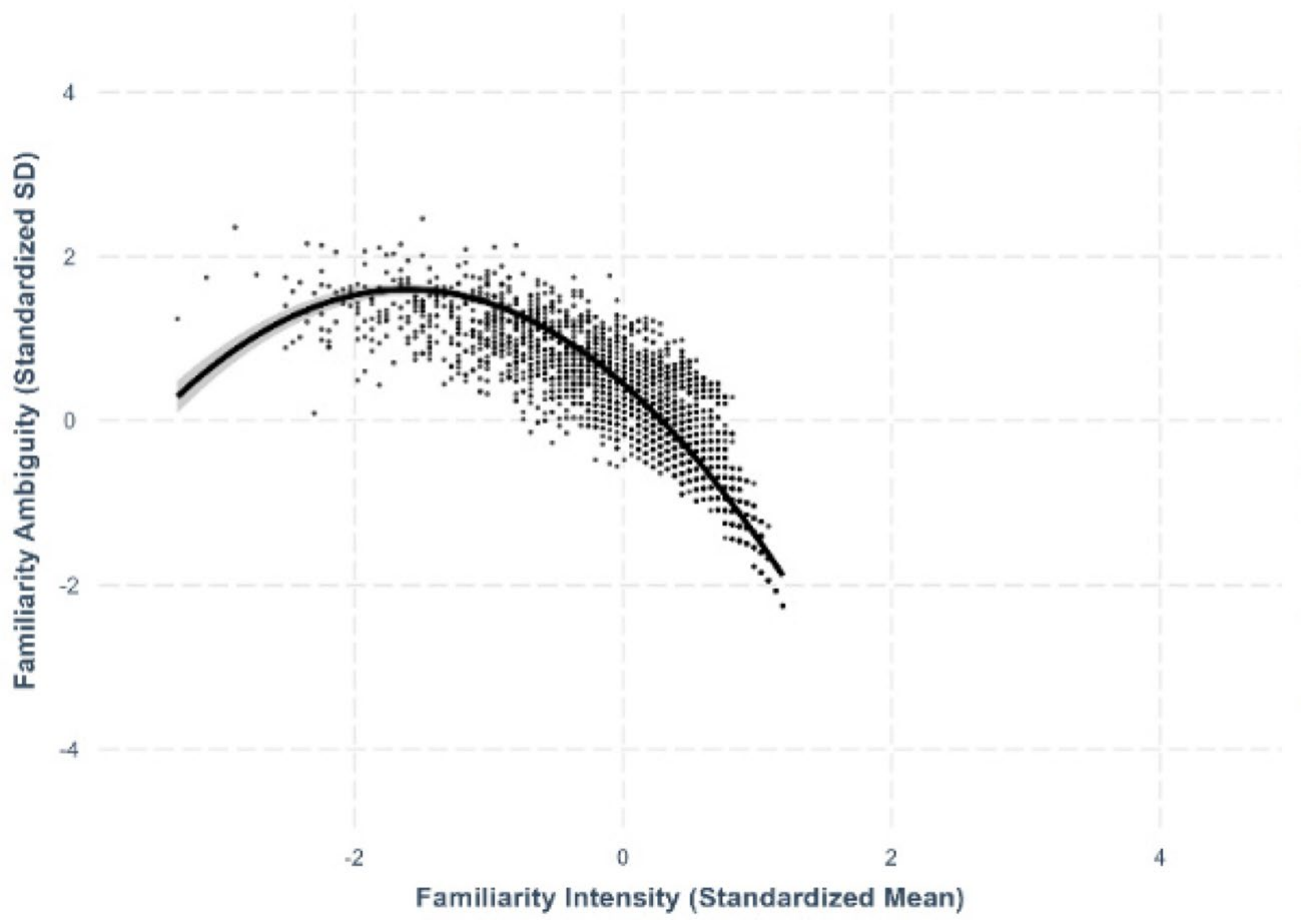
Familiarity–familiarity ambiguity

**Fig. 16:**
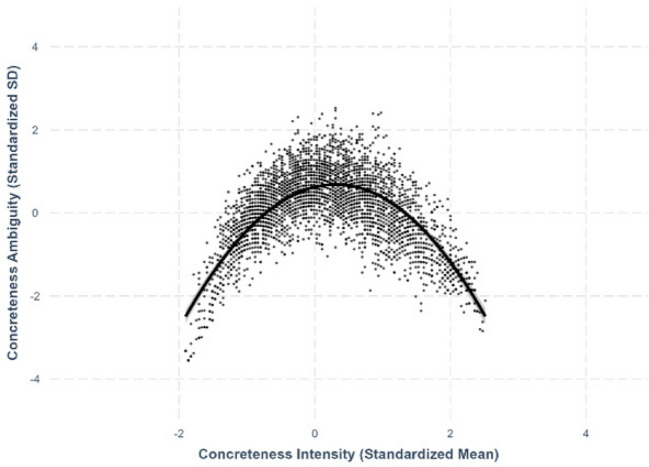
Concreteness–concreteness ambiguity

We conducted multiple regression analyses to examine the relationships with other variables (arousal, familiarity, concreteness, imageability, and AoA) as outcome variables. Each model controlled for the influence of other lexico-semantic variables and their ambiguities, which to our knowledge, was not done in some previous studies (e.g., Peng et al., 2024; Xue et al., 2024). All variables were standardized, the regression coefficients are reported in Table 2, and the adjusted R^2^ values for each model are reported below. For models where arousal and AoA are the outcome variables, adding the squared valence term significantly improved the model fit; while for models where familiarity, concreteness, and imageability are the outcome variables, adding the squared valence term did not significantly improve the model fit. The complete model statistics can be found on https://osf.io/kh4yx, and the best-fit model statistics are shown in Table 2. The relationships discussed in this section are presented in Figs. 2, 3, 4, 5, 6, 7, 8, 9, 10, 11, 12, 13, 14, 15 and 16.

**Table 2:** Standardized regression coefficients and standard error of characters’ lexical variables’ interrelationships models (*N* = 3971)

Predictor Variable	Outcome variable				
	Arousal	Familiarity	Concreteness	Imageability	Age of acquisition
Valence	–.291*** (.0147)	.0133 (.00712)	–.0279*** (.00821)	.00614 (.00811)	–.0486*** (.00973)
Valence^2^	.194*** (.0109)	---	---	---	–.0227** (.00716)
Arousal	---	.0699*** (.00833)	–.0358*** (.00974)	.0283** (.00961)	.0985*** (.0121)
Familiarity	.338*** (.0433)	---	–.113*** (.228)	.119*** (.0224)	–.749*** (.0228)
Concreteness	–.0115** (.0379)	–.0846*** (.0171)	---	.844*** (.0101)	–.0878*** (.0235)
Imageability	.104** (.0384)	.0918 (.0173)	.869*** (.0104)	---	–.0389 (.0239)
Age of acquisition	.256*** (.0315)	–.397*** (.0121)	–.0608*** (.0166)	–.0270 (.0164)	---
Valence ambiguity	.158*** (.0137)	.00284 (.00632)	–.0115 (.00730)	.0109 (.00720)	.00539 (.00871)
Arousal ambiguity	.404*** (.0153)	–.0406*** (.00771)	–.00997 (.00895)	.00916 (.00883)	–.0188 (.0107)
Familiarity ambiguity	.0106 (.0292)	–.413*** (.0104)	–.0977*** (.0151)	.0576*** (.0150)	.0948*** (.0181)
Concreteness ambiguity	.00138 (.0173)	.0197* (.00780)	.0381*** (.00900)	–.0544*** (.00883)	.0201 (.0107)
Imageability ambiguity	–.0133 (.0176)	.0286*** (.00792)	–.0977*** (.00896)	.101*** (.00882)	.0366*** (.0109)
Age of Acquisition ambiguity	.0285 (.0164)	–.0114 (.00739)	.00398 (.00854)	.0172* (.00841)	–.111*** (.00992)
Semantic radical transparency	.0517** (.0185)	–.0272** (.00838)	.0427*** (.00966)	–.0621*** (.00948)	.0443*** (.0115)
Model statistics					
Adjusted *R*^2^	.510	.821	.844	.844	.740
F-statistics	191.1***	902.5***	1064***	1063***	521.1***
Degrees of freedom	14, 2547	13, 2548	13, 2548	13, 2548	14, 2547

*Note*. ***p <.001, **p <.01, *p <.05

### Valence and other lexico-semantic variables

#### Valence-arousal

The relationship between valence and arousal was analyzed using both linear and quadratic models. The linear model revealed a significant association between the two variables (*β* = –.327, *SE* =.0154, *p* <.001). The quadratic model (best-fit model) revealed an asymmetric relationship: extremely negative characters were rated as more arousing than extremely positive characters (*β* =.194, *SE* =.0109, *p* <.001; Fig. 2).

#### Valence-familiarity

The relationship between valence and familiarity was not significant in either the linear model (*β* =.0133, *SE* =.00712, *p* =.0620) or the quadratic model (*β* =.00151, *SE* =.00523, *p* =.773). Adding the squared valence term did not significantly improve the model. Thus, character valence is not related to character familiarity (Fig. 2).

#### Valence-concreteness

We observed a weak negative relationship between valence and concreteness, as shown in Fig. 4, where more positive characters were rated as less concrete (*β* = –.0279, *SE* =.00821, *p <.*001). As the model without the squared valence term is the best-fit model, the valence–concreteness relationship is best described by a linear model (Fig. 2).

#### Valence–imageability

The association between valence and imageability was not significant in the linear valence model (*β* =.00614, *SE* =.00811, *p* =.449; see Fig. 2) or the model with squared valence term (*β* =.00123, *SE* =.00595, *p* =.836). These suggest that valence does not predict imageability, even when accounting for potential nonlinear relationships.

#### Valence–AoA

A weak asymmetric relationship was found between valence and AoA, where extremely negative characters were rated as being acquired later in life than extremely positive characters (*β* = –.0227, *SE* =.00716, *p <.*01), as depicted in Fig. 2. As the model with the squared valence term is the best-fit model, it suggests the curvilinear valence–AoA relationship.

### Arousal and other lexico-semantic variables

#### Arousal–familiarity

We found a positive linear arousal–familiarity relationship, with more arousing characters rated as more familiar (*β* =.0699, *SE* =.00834, *p <.*001; Fig. 3).

#### Arousal–concreteness

We obtained a negative linear relationship between arousal and concreteness (*β* = –.0358, *SE* =.00974, *p <.*001), indicating that the more arousing characters were rated as less concrete, as shown in Fig. 2.

#### Arousal–imageability

We observed a positive linear relationship between arousal and imageability, where more arousing characters were rated as more imageable (*β* =.0283, *SE* =.00961, *p <.*01; Fig. 3).

#### Arousal–AoA

We found a positive linear relationship between arousal and AoA, where more arousing characters were rated as acquired later in life (*β* =.0985, *SE* =.0121, *p <.*001; Fig. 4).

### Relationships among lexico-semantic variables

#### Familiarity–concreteness

We obtained a negative linear relationship between familiarity and concreteness, suggesting that more familiar characters were rated as less concrete (*β* = –.113, *SE* =.0223, *p <.*001; Fig. 4).

#### Familiarity–imageability

We obtained a positive linear relationship between familiarity and imageability, with more familiar characters rated as more imageable (*β* =.119, *SE* =.224, *p <.*001; Fig. 5).

#### Familiarity–AoA

We found a negative linear relationship between familiarity and AoA, where more familiar characters were rated as being acquired earlier in life (*β* = –.749, *SE* =.0228, *p <.*001; Fig. 6).

#### Concreteness–imageability

We found a strong positive linear relationship between concreteness and imageability, where more concrete characters were rated as more imageable (*β* =.844, *SE* =.0101, *p <.*001; Fig. 7).

#### Concreteness–AoA

A negative linear relationship was observed between concreteness and AoA, with more concrete characters rated as being acquired earlier in life (*β* = –.0878, *SE* =.0235, *p <.*001; Fig. 8).

#### Imageability–AoA

No significant relationship was found between imageability and AoA, indicating that the imageability of a character did not predict its AoA (*β* = –.0389, *SE* =.0239, *p* =.103; Fig. 9).

### Valence ambiguity and other lexico-semantic variables

To examine how valence ambiguity is associated with other lexico-semantic variables, we conducted regression analyses with valence ambiguity as the outcome variable, controlling for all other lexico-semantic variables and their ambiguities. The regression coefficients are shown in Table 3 and the relationships are shown on Fig. 10. Model comparison using ANOVA indicated that including the squared valence term (*R*^*2*^ =.0743, *F*(14,2547) = 15.7, *p <.*001) significantly improved the model fit for valence ambiguity compared to when only the linear valence term was included. The complete statistics for the latter model can be found on https://osf.io/kh4yx.

**Table 3:** Standardized regression coefficients and standard errors for intensity–ambiguity regression models in Chinese characters

Predictor variable	Outcome variable					
	Valence ambiguity	Arousal ambiguity	Familiarity ambiguity	Concreteness ambiguity	Imageability ambiguity	Age-of-acquisition ambiguity
Valence	.174*** (.0220)	.0535** (.0166)	.00132 (.00851)	–.0190 (.0131)	.0231 (.0145)	–.0511** (.0187)
Valence^2^	–.0654*** (.0163)	---	---	---	---	---
Arousal	.316*** (.0273)	.753*** (.0184)	.0160 (.0101)	–.0432** (.0157)	–.0523** (.0172)	.0299 (.0222)
Arousal^2^	---	–.247*** (.0109)	---	---	---	---
Familiarity	.0291 (.0619)	–.233*** (.0459)	−1.431*** (.0228)	–.151*** (.0370)	–.109** (.0409)	–.479*** (.0648)
Familiarity^2^	---	---	–.445*** (.0116)	---	---	---
Concreteness	–.0897 (.0536)	–.0612 (.0400)	–.118*** (.0204)	.399*** (.0319)	–.204*** (.0348)	.0336 (.0451)
Concreteness^2^	---	---	---	–.652*** (.0137)	---	---
Imageability	.0828 (.0543)	.0844* (.0406)	.0370 (.0208)	–.142*** (.0320)	.435*** (.0345)	.0663 (.0457)
Imagaeability^2^	---	---	---	---	–.617*** (.0171)	---
Age of acquisition	.0279 (.0451)	–.116*** (.0336)	–.194*** (.0189)	–.122*** (.0269)	–.0838** (.0297)	–.656*** (.0436)
Age of acquisition^2^	---	---	---	---	---	–.248*** (.0242)
Valence ambiguity	---	.000313 (.0147)	.00955 (.00755)	.00408 (.0117)	–.0239 (.0128)	–.0109 (.0166)
Arousal ambiguity	.00173 (.0243)	---	–.0159 (.00925)	.00669 (.0143)	.0311* (.0157)	–.0429* (.0204)
Familiarity ambiguity	.0336 (.0413)	–.0358 (.0308)	---	–.0507* (.0244)	.0575* (.0269)	.0921* (.0367)
Concreteness ambiguity	.0346 (.0244)	–.0187 (.0182)	–.0111 (.00934)	---	.000592 (.0176)	–.0440* (.0207)
Imageability ambiguity	–.0344 (.0248)	.0184 (.0185)	.0130 (.00951)	.0774*** (.0149)	---	–.0259 (.0208)
Age of acquisition ambiguity	–.0277 (.0231)	–.0569*** (.00172)	–.0113 (.00901)	–.00561 (.0136)	–.0207 (.0150)	---
Semantic radical transparency	–.0411 (.0262)	–.0270 (.0196)	.00848 (.0100)	.0130 (.0155)	–.0261 (.0171)	.0704** (.0220)
Model statistics						
Adjusted *R*^2^	.0743	.431	.835	.541	.459	.135
F-statistics	15.7***	139.5***	929.1***	216.9***	156***	29.44***
Degrees of freedom	14, 2547	14, 2547	14, 2547	14, 2547	14, 2547	14, 2547

*Note*. ***p <.001, **p <.01, *p <.05

The best-fit model revealed a significant positive arousal–valence ambiguity linear relationship (*β* =.316, *SE* =.0273, *p <.*001; see Fig. 10), indicating that more arousing characters exhibited greater variability in perceived valence. However, the associations of valence ambiguity with familiarity (*β* =.0291, *SE* =.0619, *p* =.638; Fig. 10), concreteness (*β* = –.0897, *SE* =.0536, *p* =.094; Fig. 10), imageability (*β* =.0828, *SE* =.0543, *p* =.128; Fig. 10), and AoA (*β* =.0279, *SE* =.0451, *p* =.536; Fig. 10) were nonsignificant. While familiarity and AoA exhibited a positive trend and concreteness and AoA showed a negative trend, these nonsignificant relationships suggest that among affective and lexico-semantic variables, arousal is the only variable that consistently predicts valence ambiguity.

### Ambiguity as moderator for valence–arousal relationship

To test whether valence ambiguity and arousal ambiguity moderate the valence–arousal relationship as shown in previous studies (Brainerd, 2018), we extended the best-fit model predicting arousal (Model-1b) by adding interaction terms. Specifically, we included the interaction between valence and valence ambiguity in Model-1a (*R*^*2*^ =.510, *F*(16,2545) = 167.6, *p <.*001) and between valence and arousal ambiguity in Model-1b (*R*^*2*^ =.541, *F*(16,2545) = 189.8, *p <.*001). The regression coefficients are summarized in Table 4.

**Table 4:** Standardized regression coefficients and standard error for arousal models

Predictor variable	Arousal as outcome variable	
	Model-1a	Model-1b
Valence	–.293*** (.0148)	–.336*** (.0154)
Valence^2^	.199*** (.0112)	.259*** (.0120)
Familiarity	.337*** (.0433)	.335*** (.0419)
Concreteness	–.116** (.0379)	–.102** (.0367)
Imageability	.105** (.0384)	.0945* (.0372)
Age of acquisition	.258*** (.0315)	.253*** (.0305)
Valence ambiguity	.142*** (.0167)	.155*** (.0132)
Arousal ambiguity	.402*** (.0153)	.499*** (.0173)
Familiarity ambiguity	.00900 (.0293)	.00451 (.0283)
Concreteness ambiguity	.00149 (.0173)	.00801 (.0168)
Imageability ambiguity	–.0133 (.0176)	–.0145 (.0170)
Age of acquisition ambiguity	.0291 (.0164)	.0244 (.0158)
Semantic radical transparency	.0512** (.0185)	.0502** (.0179)
Valence × valence ambiguity	–.0116 (.0144)	---
Valence^2^ × valence ambiguity	.0186 (.104)	---
Valence × arousal ambiguity	---	.0940*** (.0144)
Valence^2^ × arousal ambiguity	---	–.110*** (.0102)
Model statistics		
Adjusted *R*^2^	.510	.541
F-statistics	167.6***	189.8***
Degrees of freedom	16, 2545	16, 2545

*Note*. Model-1a includes valence ambiguity, Model-1b includes arousal ambiguity. ***p <.001, **p <.01, *p <.05

The interaction terms for valence × valence ambiguity (*β* = –.0116, *SE* =.0144, *p* =.422) and valence^2^ × valence ambiguity (*β* =.0186, *SE* =.0104, *p* =.0741) in Model-1a were nonsignificant, suggesting that valence ambiguity did not moderate the effect of valence on arousal, as shown in Fig. 11. In contrast, the interaction terms for valence × arousal ambiguity (*β* =.0940, *SE* =.0144, *p <.*001) and valence^2^ × arousal ambiguity (*β* = –.110, *SE* =.0102, *p <.*001) in Model-1b were significant, indicating that arousal ambiguity moderated the valence–arousal relationship, as shown in Fig. 12. Specifically, when arousal ratings are less variable, extreme valence leads to higher arousal; conversely, when arousal ratings are highly variable, extreme valence is associated with lower arousal.

### Intensity–ambiguity relationship

**Fig. 17:**
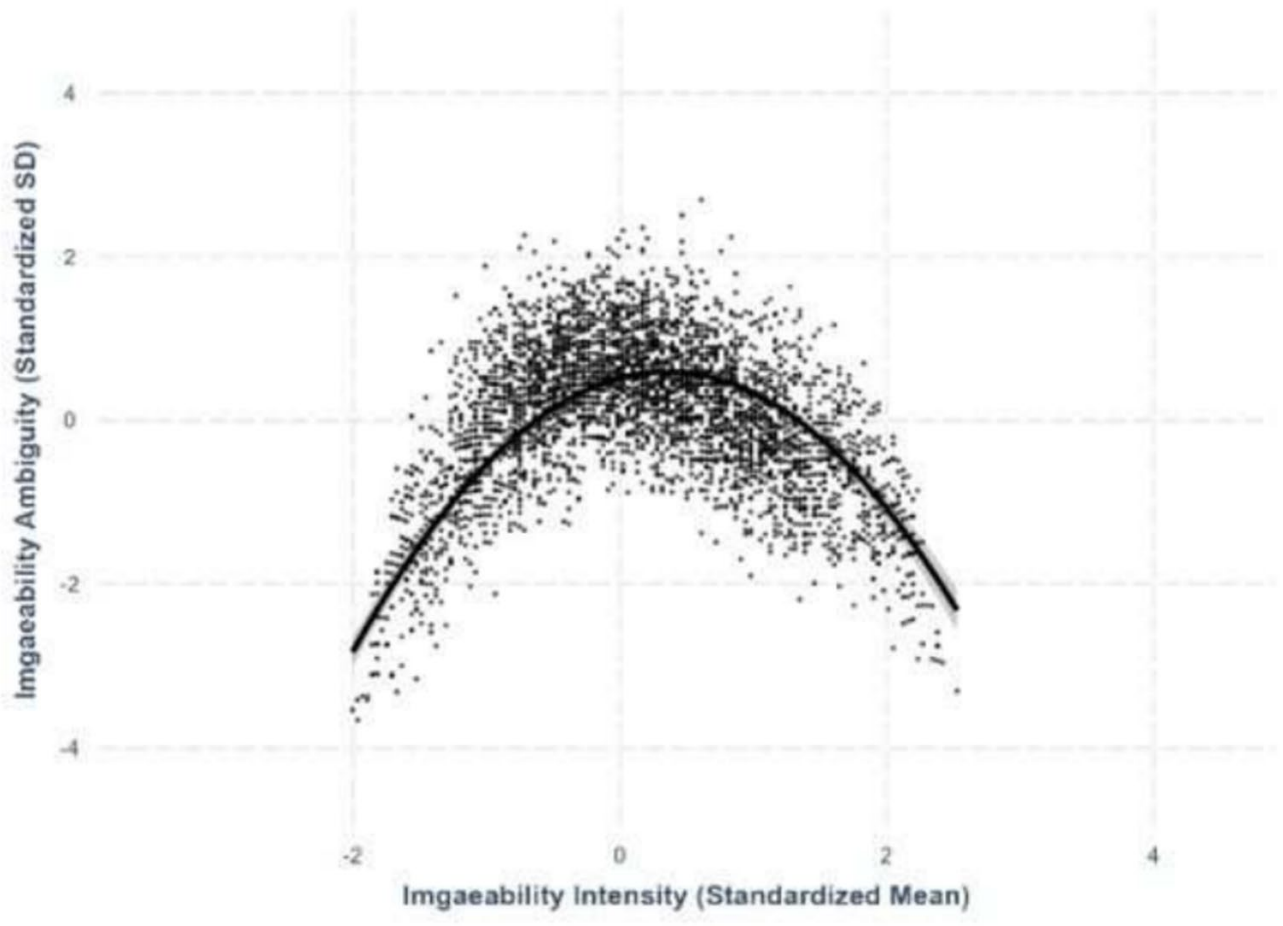
Imageability–imageability ambiguity

**Fig. 18:**
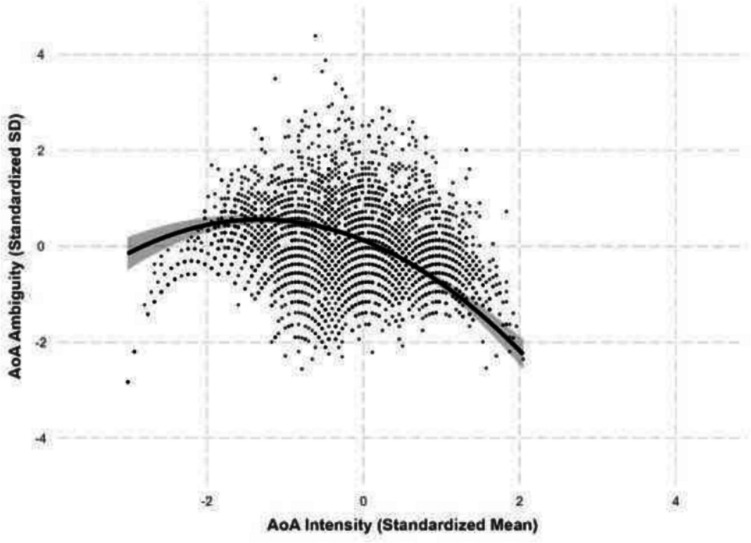
Age of acquisition–age of acquisition ambiguity

Following Brainerd et al. (2021a, b) and Chan and Tse (2024), we investigated the quadratic intensity–ambiguity relationship for valence, arousal, familiarity, concreteness, imageability, and AoA of Chinese characters. All lexico-semantic variables and their ambiguities are controlled. For each ambiguity as the outcome variable, we first examined the linear intensity–ambiguity pattern and then added a squared intensity term to test for a quadratic pattern. For all models, adding a squared intensity term significantly improved the model fit. All best-fit model results are summarized in Table 3, and the intensity–ambiguity relationships are shown in Figures 13, 14, 15, 16, 17 and 18. The complete model statistics are shown on https://osf.io/kh4yx.

### Valence–valence ambiguity

We observed an asymmetric quadratic relationship between valence and valence ambiguity, where extremely positive characters exhibited greater variability in perceived valence than extremely negative characters (*β* = –.0654, *SE* =.0163, *p <.*001; see Fig. 13).

### Arousal–arousal ambiguity

We found an asymmetric quadratic relationship between arousal and arousal ambiguity, where extremely arousing characters exhibited greater variability in perceived arousal than least arousing characters (*β* = –.247, *SE* =.0109, *p <.*001; see Fig. 14).

### Familiarity–familiarity ambiguity

We identified an asymmetric quadratic relationship between familiarity and its ambiguity, where extremely familiar characters exhibited less variability in perceived familiarity than less familiar characters (*β* = –.445, *SE* =.0116, *p <.*001; see Fig. 15).

### Concreteness–concreteness ambiguity

We found a quadratic relationship between concreteness and concreteness ambiguity, where both extremely concrete and extremely abstract characters exhibited lower ambiguity compared to moderately concrete characters (*β* = –.652, *SE* =.0137, *p <.*001; see Fig. 16).

### Imageability–imageability ambiguity

We identified a quadratic relationship between imageability and imageability ambiguity, where highly imageable and least imageable characters exhibited lower ambiguity compared to moderately imageable characters (*β* = –.617, *SE* =.0171, *p <.*001; see Fig. 17).

### AoA–AoA ambiguity

We obtained an asymmetric quadratic relationship between AoA and AoA ambiguity, where characters rated as acquired later exhibited less variability in AoA compared to characters rated as being acquired earlier in life (*β* = –.248, *SE* =.0242, *p <.*001; see Fig. 18).

### Character-level and word-level relationship

To examine the relationship between character-level and word-level variables, we matched the first and second characters with the corresponding two-character words and conducted multiple regressions with word-level variables being outcome variables and character-level lexico-semantic variables being predictor variables. In each of the analyses, those word-level variables that were not the outcome variable were controlled. For example, when word valence was the outcome variable, word arousal, familiarity, concreteness, and imageability were all controlled. Due to the absence of AoA data at the word level, we focused on word valence, arousal, familiarity, concreteness, and imageability in these analyses. Model comparison using ANOVA indicated that adding squared character valence terms significantly improved the model fit for all outcome variables. The complete model statistics can be found on https://osf.io/kh4yx. In another set of analyses, we also included the character variable × character semantic transparency interaction term to test the potential moderating role of character semantic transparency in these relationships.

**Fig. 19:**
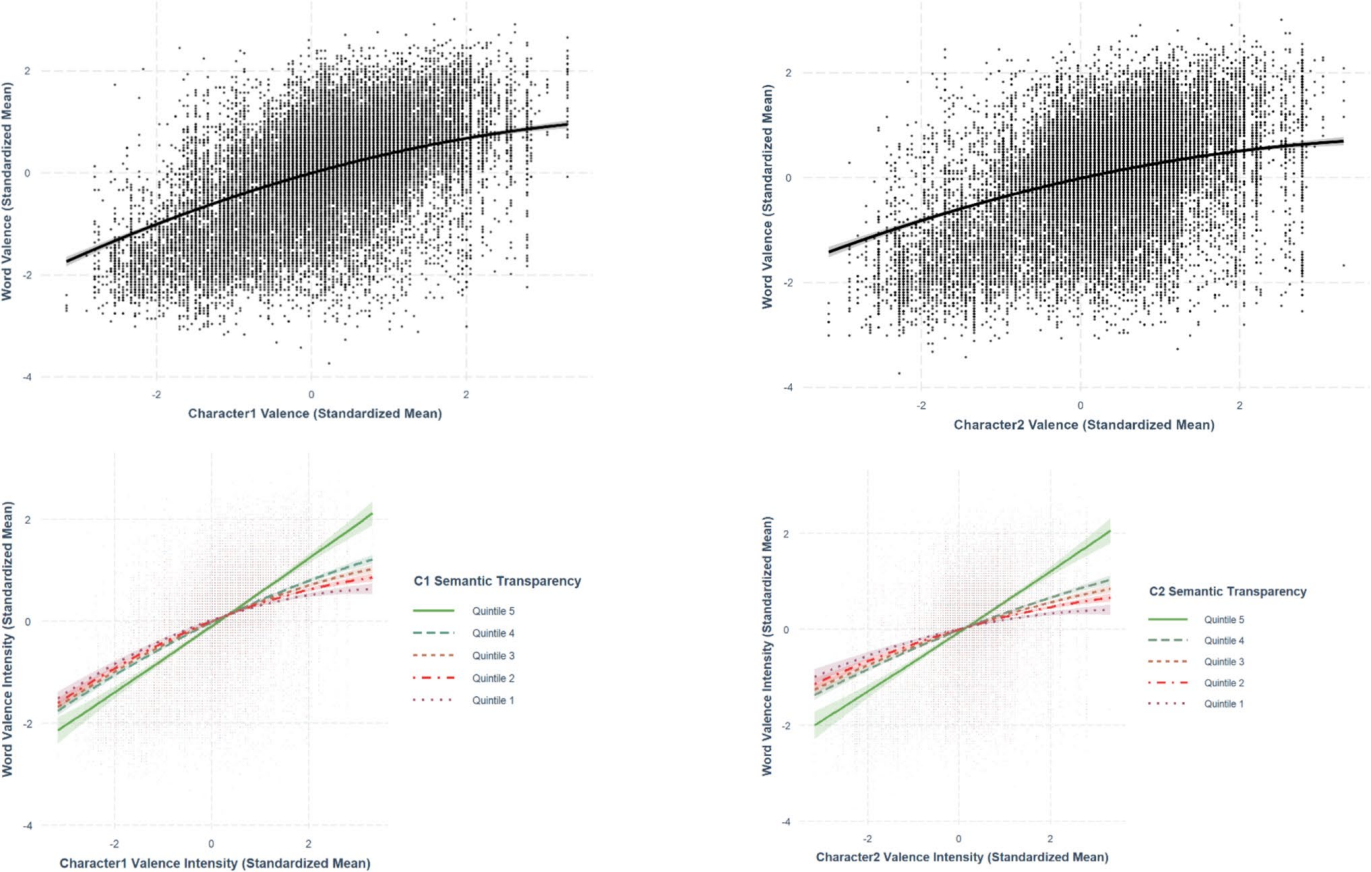
Character valence–word valence relationship. *Note.* The two figures at the bottom present the moderation effect of character semantic transparency in the character valence–word valence relationship

**Fig. 20:**
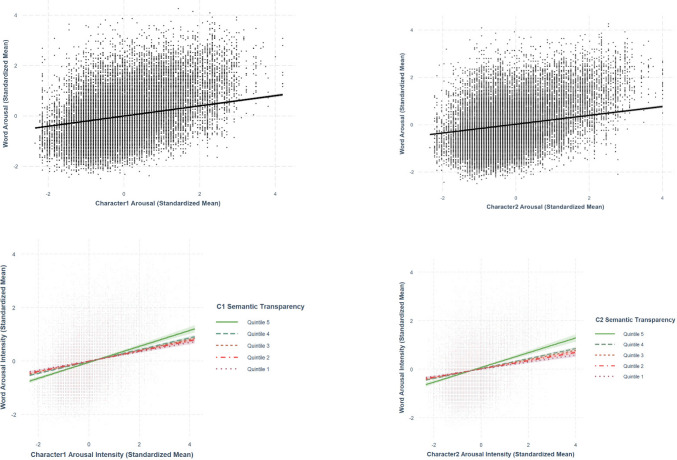
Character arousal–word arousal. *Note.* The two figures at the bottom present the moderation effect of character semantic transparency in the character arousal–word arousal relationship

**Fig. 21:**
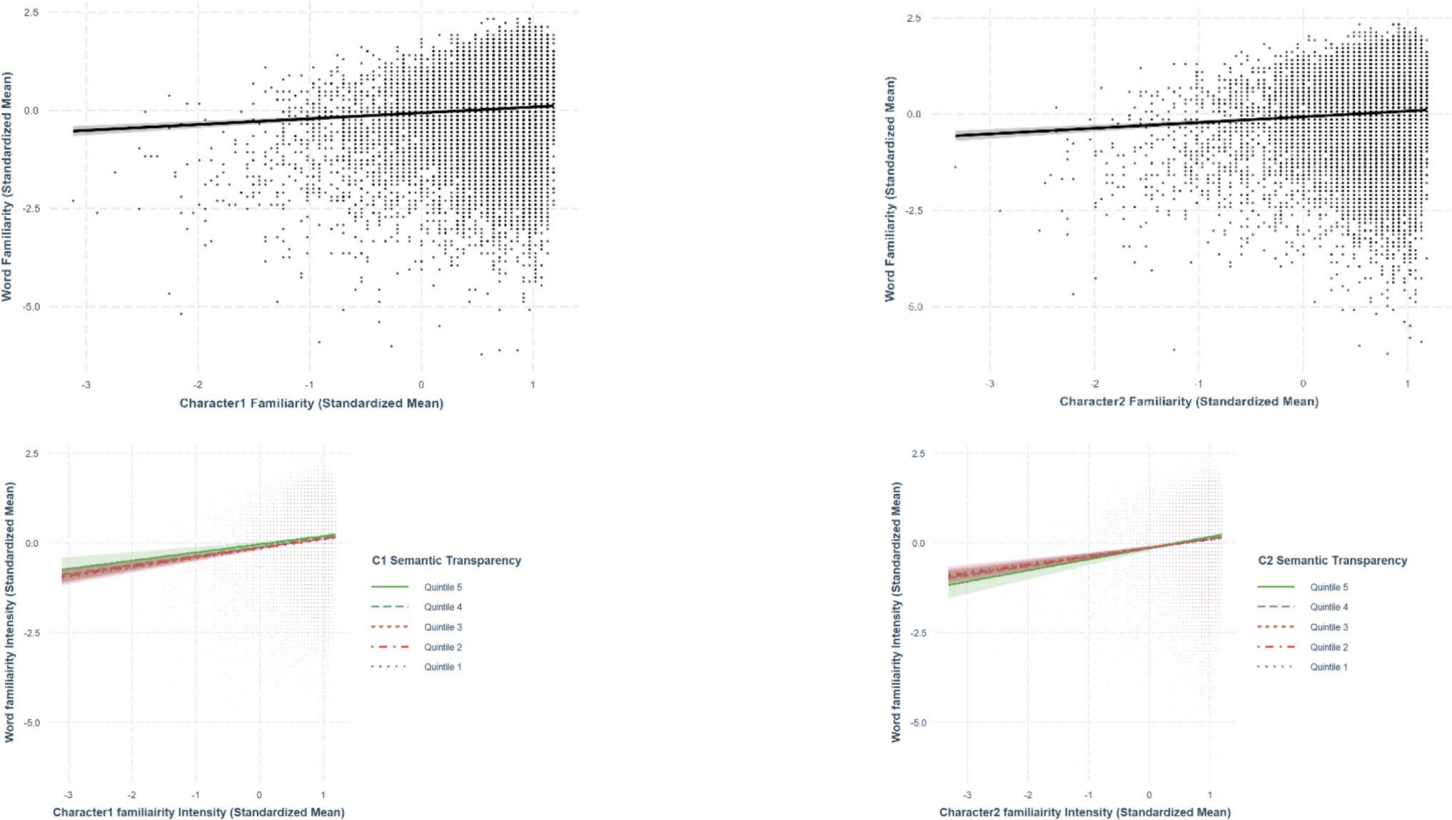
Character familiarity–word familiarity. *Note.* The two figures at the bottom present the moderation effect of character semantic transparency in the character familiarity–word familiarity relationship

**Fig. 22:**
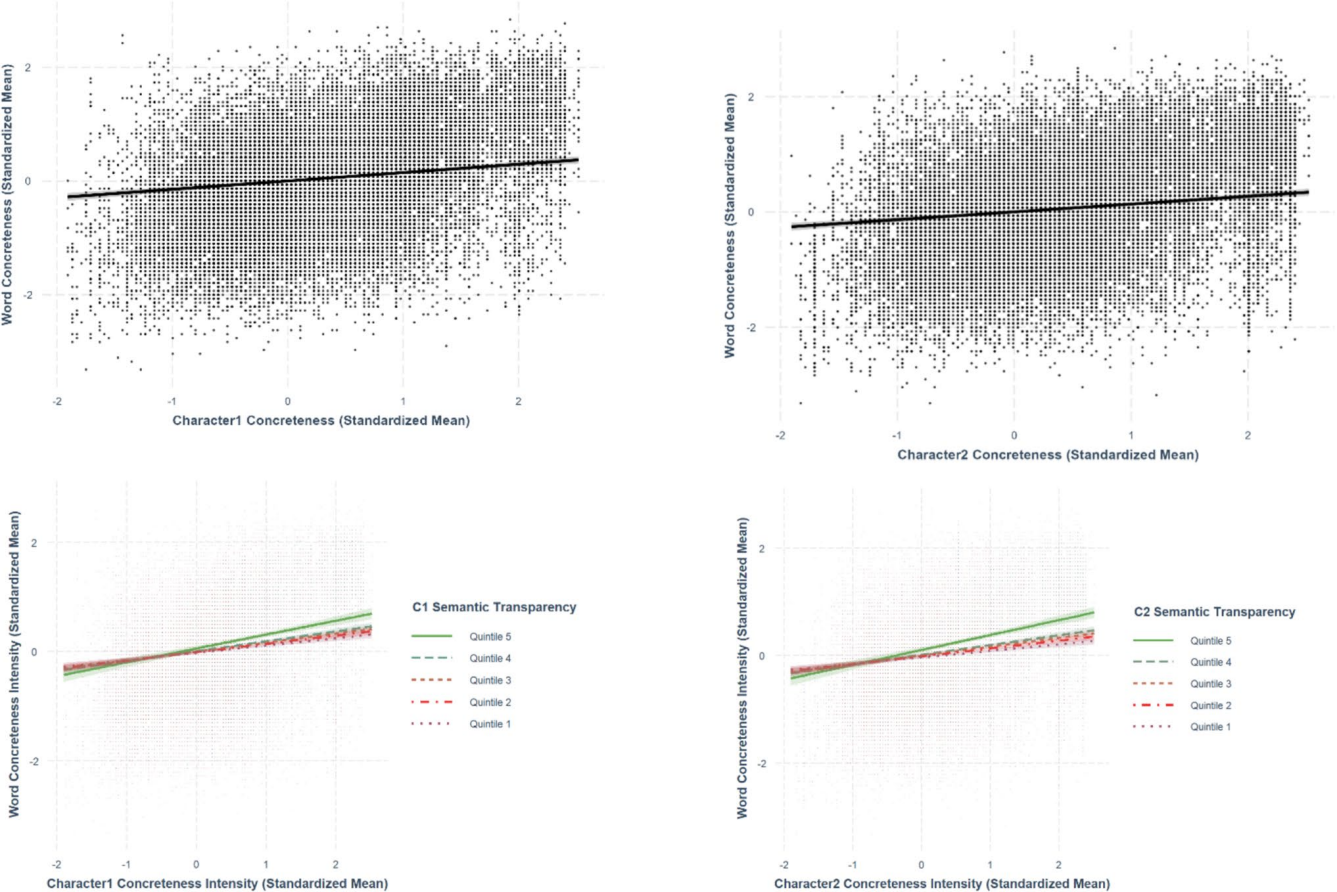
Character concreteness–word concreteness. *Note.* The two figures at the bottom present the moderation effect of character semantic transparency in the character concreteness–word concreteness relationship

**Fig. 23:**
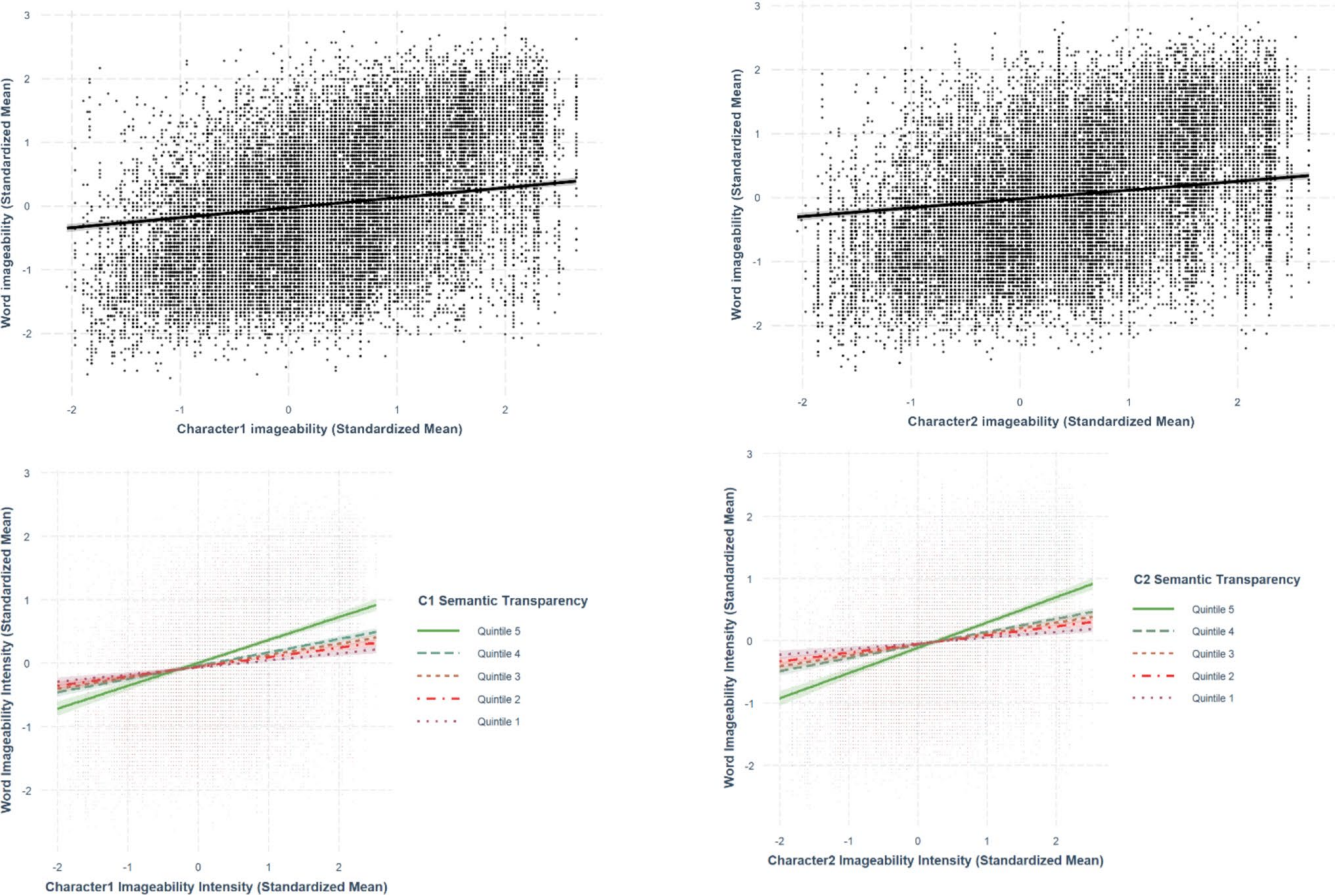
Character imageability–word imageability. *Note.* The two figures at the bottom present the moderation effect of character semantic transparency in the character imageability–word imageability relationship

Only best-fit models are presented in the paper. The two sets of analyses are summarized in Tables 5 and 6, respectively. The relationships discussed in this section are presented from Figs. 19, 20, 21, 22, and 23.

**Table 5:** Standardized regression coefficients and standard errors for character-level and word-level lexical variables

Predictor variable	Outcome variable				
	Word valence	Word arousal	Word familiarity	Word concreteness	Word imageability
C1 Valence	.421*** (.00611)	–.0150* (.00590)	–.0118* (.00520)	.00993 (.00679)	–.00632 (.00621)
C1 Valence^2^	–.0396*** (.00401)	.0115*** (.00344)	.00510 (.00303)	-.0142*** (.00395)	–.0116** (.00362)
C1 Arousal	.0667*** (.00804)	.202*** (.00672)	.0157** (.00608)	–.0107 (.00793)	–.0129 (.00726)
C1 Familiarity	.00943 (.0317)	–.0184 (.0272)	.254*** (.0239)	.0998** (.0313)	–.0311 (.0286)
C1 Concreteness	–.0575*** (.0158)	–.0261 (.0135)	–.00316 (.0119)	.150*** (.0155)	.0451** (.0142)
C1 Imageability	.0151 (.0148)	.0257* (.0126)	–.0128 (.0112)	–.0220 (.0145)	.157*** (.0133)
C1 Age of acquisition	–.0229* (.0111)	.0786*** (.00944)	–.0229** (.00834)	.0218* (.0109)	–.00539 (.00996)
C1 Semantic transparency	.00634 (.00576)	–.0153** (.00493)	.0219*** (.00435)	.0337*** (.00567)	.0480*** (.00518)
C1 Valence ambiguity	.0287*** (.00540)	.0184*** (.00463)	.000551 (.00408)	–.00323 (.00658)	.00403 (.00488)
C1 Arousal ambiguity	–.0223*** (.00668)	–.00892 (.00571)	.00256 (.00504)	.00376 (.00658)	.00455 (.00602)
C1 Familiarity ambiguity	–.0286 (.0120)	.0110 (.0102)	.0529*** (.00901)	.0112 (.0118)	–.00824 (.0108)
C1 Concreteness ambiguity	.00455 (.00697)	.000259 (.00596)	–.00761 (.00526)	–.0130 (.00686)	–.00491 (.00628)
C1 Imageability ambiguity	.0170* (.00674)	–.00261 (.00577)	.00504 (.00509)	.00793 (.00664)	–.00593 (.00608)
C1 Age of acquisition ambiguity	–.00215 (.00661)	–.0128* (.00566)	–.00277 (.00499)	–.00431 (.00651)	.0111 (.00596)
C2 Valence	.330*** (.00649)	–.0248*** (.00595)	–.0177*** (.00525)	–.00629 (.00685)	–.0186** (.00627)
C2 Valence^2^	–.0351*** (.00423)	.0186*** (.00363)	.0102** (.00320)	.0280*** (.00417)	–.0168*** (.00382)
C2 Arousal	.0336*** (.00821)	.186*** (.00688)	.00780 (.00620)	–.0259** (.00808)	–.0207** (.00740)
C2 Familiarity	.0352 (.0315)	–.0471 (.0269)	.245*** (.0237)	.131*** (.0310)	–.0508 (.0284)
C2 Concreteness	–.0693*** (.0158)	–.00210 (.0135)	–.00361 (.0119)	.142*** (.0155)	.0772*** (.0142)
C2 Imageability	.0421** (.0148)	.0243 (.0127)	–.00732 (.0112)	–.0232 (.0146)	.147*** (.0133)
C2 Age of acquisition	–.00942 (.0114)	.0621*** (.0974)	.0100 (.00860)	.0258* (.0112)	–.000949 (.0103)
C2 Semantic transparency	.0269*** (.00585)	.00693 (.00501)	.0143** (.00442)	.0492*** (.00575)	.0169** (.00527)
C2 Valence ambiguity	.0295*** (.00525)	.0262*** (.00449)	.00511 (.00397)	.000308 (.00517)	–.00371 (.00474)
C2 Arousal ambiguity	–.00747 (.00687)	–.0127* (.00587)	–.000817 (.00518)	.0155* (.00676)	–.00823 (.00619)
C2 Familiarity ambiguity	.0176 (.0120)	.0187 (.0103)	.0524*** (.00908)	.0121 (.0119)	–.000531 (.0109)
C2 Concreteness ambiguity	.00951 (.00705)	.0202*** (.00603)	.00129 (.00532)	.000158 (.00694)	–.0149* (.00635)
C2 Imageability ambiguity	.0000662 (.00654)	–.0105 (.00559)	–.00652 (.00494)	.00533 (.00644)	.00845 (.00589)
C2 Age of acquisition ambiguity	.00152 (.00680)	–.0128* (.00582)	.00943 (.00514)	.00157 (.00670)	.0155* (.00613)
Model statistics					
Adjusted *R*^2^	.542	.653	.666	.545	.621
F-statistics	516.4***	822.5***	868.9***	523.2***	714.2***
Degrees of freedom	40, 17398	40, 17398	40, 17398	40, 17398	40, 17398

Note.***p <.001, **p <.01, *p <.05

**Table 6:** Standardized regression coefficients and standard deviation for character-level and word-level correlation moderated by semantic transparency

Predictor variable	Outcome variable				
	Word valence	Word arousal	Word familiarity	Word concreteness	Word imageability
C1 Valence	.399*** (.00624)	–.0112 (.00590)	–.0119* (.00520)	.0111 (.00677)	–.00403 (.00615)
C1 Valence^2^	–.0368*** (.00413)	.00939** (.00344)	.00503 (.00303)	–.0136*** (.00395)	–.0109** (.00358)
C1 Arousal	.0622*** (.00791)	.196*** (.00673)	.0155* (.00608)	–.00848 (.00792)	–.00875 (.00719)
C1 Familiarity	.00618 (.0313)	–.0188 (.0271)	.257*** (.0241)	.0964** (.0312)	–.0412 (.0283)
C1 Concreteness	–.0474** (.0156)	−0.0194 (.0135)	–.00306 (.0119)	.157*** (.0155)	.0609*** (.0141)
C1 Imageability	–.00372 (.0146)	0.0240 (.0126)	–.0128 (.0111)	–.0179 (.0145)	.160*** (.0131)
C1 Age of acquisition	–.0178 (.0109)	0.0823*** (.00942)	–.0227** (.00835)	.0293** (.0109)	–.0105 (.00989)
C1 Semantic transparency	–.0279*** (.00687)	−0.0118* (.00496)	.0293** (.0103)	.0193** (.00643)	.0177** (.00576)
C1 Valence ambiguity	.0304*** (.00532)	.0163*** (.00462)	.000492 (.00408)	–.00409 (.00532)	.00247 (.00482)
C1 Arousal ambiguity	–.0204** (.00658)	–.00608 (.00571)	.00254 (.00504)	.00290 (.00657)	.00302 (.00596)
C1 Familiarity ambiguity	–.00452 (.0118)	.0133 (.0102)	.0537*** (.00904)	.00927 (.0117)	–.0134 (.0107)
C1 Concreteness ambiguity	.00525 (.00686)	.00574 (.00595)	–.00797 (.00528)	–.00684 (.00695)	.00551 (.00628)
C1 Imageability ambiguity	.0114 (.00664)	–.00254 (.00575)	.00479 (.00509)	.0118 (.00665)	.00566 (.00607)
C1 Age of acquisition ambiguity	–.00505 (.00651)	–.0120* (.00564)	–.00270 (.00499)	–.00619 (.00651)	.00647 (.00590)
C2 Valence	.295*** (.00703)	−0.0167** (.00602)	–.0180*** (.00525)	–.00594 (.00684)	–.0192** (.00620)
C2 Valence^2^	–.0239*** (.00452)	.0148*** (.00365)	.0103** (.00320)	–.0270*** (.00417)	–.0145*** (.00378)
C2 Arousal	.0356*** (.00809)	.175*** (.00701)	.00836 (.00621)	–.0236** (.00806)	–.0216** (.00732)
C2 Familiarity	.0419 (.0310)	–.0492 (.0268)	.239*** (.0239)	.122*** (.0310)	–.0656* (.0281)
C2 Concreteness	–.0609*** (.0156)	–.00600 (.0135)	–.00468 (.0120)	.151*** (.0155)	.0943*** (.0141)
C2 Imageability	.0338* (.0146)	.0243 (.0126)	–.00762 (.0112)	–.0178 (.0146)	.150*** (.0132)
C2 Age of acquisition	–.00615 (.0112)	.0646*** (.00972)	.00947 (.00861)	.0282* (.0112)	–.00553 (.0102)
C2 Semantic transparency	–.0154* (.00684)	.0168** (.00519)	–.00200 (.0105)	.0317*** (.00648)	–.0161** (.00580)
C2 Valence ambiguity	.0285*** (.00518)	.0261*** (.00448)	.00476 (.00397)	.000780 (.00516)	–.00198 (.00486)
C2 Arousal ambiguity	–.00604 (.00678)	–.00772 (.00589)	–.000996 (.00518)	.0147* (.00675)	–.00732 (.00612)
C2 Familiarity ambiguity	.0169 (.0119)	.0160 (.0103)	.0511*** (.00911)	.000794 (.0119)	–.00784 (.0107)
C2 Concreteness ambiguity	.00940 (.00694)	.0222*** (.00602)	.00186 (.00533)	.00721 (.00704)	–.00265 (.00636)
C2 Imageability ambiguity	.000556 (.00645)	–.00992 (.00558)	–.00602 (.00495)	.00951 (.00646)	.0192** (.00588)
C2 Age of acquisition ambiguity	.00203 (.00670)	–.0122* (.00580)	.00915 (.00514)	–.00365 (.00673)	.00418 (.00611)
C1 Valence × C1 Semantic transparency	.0772*** (.00540)	---	---	---	---
C1 Valence^2^ × C1 Semantic transparency	.0120*** (.00362)	---	---	---	---
C2 Valence × C2 Semantic transparency	.0923*** (.00620)	---	---	---	---
C2 Valence^2^ × C2 Semantic transparency	.00778* (.00374)	---	---	---	---
C1 Arousal × C1 Semantic transparency	---	.0306*** (.00441)	---	---	---
C2 Arousal × C2 Semantic transparency	---	.0356*** (.00489)	---	---	---
C1 Familiarity × C1 Semantic transparency	---	---	–.00846 (.0118)	---	---
C2 Familiarity × C2 Semantic transparency	---	---	.0197 (.0116)	---	---
C1 Concreteness × C1 Semantic transparency	---	---	---	.0290*** (.00543)	---
C2 Concreteness × C2 Semantic transparency	---	---	---	.0353*** (.00530)	---
C1 Imageability × C1 Semantic transparency	---	---	---	---	.0602*** (.00484)
C2 Imageability × C2 Semantic transparency	---	---	---	---	.0713*** (.00481)
Model statistics					
Adjusted *R*^2^	.556	.655	.666	.547	.630
F-statistics	494***	790.6***	827.6***	502.3***	705.2
Degrees of freedom	44, 17394	42, 17396	42, 17396	42, 17396	42, 17396

*Note*. ***p <.001, **p <.01, *p <.05

### Character valence-word valence

Using best-fit model, we observed curvilinear relationships between both first-character valence (*β* =.421, *SE* =.00611, *p <.*001) and second-character valence (*β* =.330, *SE* =.00649, *p <.*001), and their corresponding word valence, such that as valence increased, its positive effect on word valence diminished (Fig. 19). Based on the magnitude of the standardized regression coefficients, the relationship between character valence and word valence was larger in the first character than in the second character. Furthermore, the interaction terms for both first-character (*β* =.0772, *SE* =.00540, *p <.*001) and second-character valence (*β* =.0923, *SE* =.00620, *p <.*001) with character semantic transparency were significant, indicating that character semantic transparency moderated the relationship between character valence and word valence. Specifically, the relationship between character valence and word valence was stronger and more linear for words with more transparent characters than those with more opaque characters (Fig. 19).

### Character arousal-word arousal

Using the best-fit model, we found positive relationships between both first-character arousal (*β* =.202, *SE* =.00672, *p <.*001) and second-character arousal (*β* =.186, *SE* =.00689, *p <.*001) with word arousal (Fig. 20). The relationship between character arousal and word arousal was larger in the first character than in the second character. The significant interaction terms between both first-character (*β* =.0306, *SE* =.00441, *p <.*001) and second-character arousal (*β* =.0356, *SE* =.00489, *p <.*001) and character semantic transparency indicated that character semantic transparency moderated the relationship between character arousal and word arousal. Specifically, the relationship was stronger for words with more transparent characters than those with more opaque characters (Fig. 20).

### Character familiarity–word familiarity

Using the best-fit model, we obtained positive relationships between both first-character familiarity (*β* =.254, *SE* =.0239, *p <.*001) and second-character familiarity (*β* =.245, *SE* =.0237, *p <.*001) with word familiarity (Fig. 21). The relationship between character familiarity and word familiarity was larger in the first character than in the second character. According to the interaction model, character semantic transparency did not moderate the relationship between word familiarity and first-character familiarity (*β* = –.00846, *SE* =.0118, *p* =.475) or between word familiarity and second-character familiarity (*β* =.0197, *SE* =.0116, *p* =.0896; see Fig. 21).

### Character concreteness–word concreteness

Using the best-fit model, we obtained positive relationships between both first-character concreteness (*β* =.150, *SE* =.0155, *p <.*001) and second-character concreteness with word concreteness (*β* =.142, *SE* =.0155, *p <.*001; see Fig. 22). The relationship between character concreteness and word concreteness was larger in the first character than in the second character. The significant interaction terms between both first-character (*β* =.0299, *SE* =.00543, *p <.*001) and second-character concreteness (*β* =.0362, *SE* =.00531, *p <.*001) and character semantic transparency indicated that the character semantic transparency moderated the relationship between character concreteness and word concreteness. Specifically, higher semantic transparency amplified the positive linear effect of character concreteness on word concreteness (Fig. 22).

### Character imageability–word imageability

Using the best-fit model, we observed positive relationships between both first-character imageability (*β* =.158, *SE* =.0133, *p <.*001) and second-character imageability (*β* =.147, *SE* =.0133, *p <.*001) with word imageability (Fig. 23). The relationship between character imageability and word imageability was larger in the first character than in the second character. The significant interaction terms between both first-character (*β* =.0602, *SE* =.00484, *p <.*001) and second-character imageability (*β* =.0713, *SE* =.00481, *p <.*001) and character semantic transparency indicated that the character semantic transparency moderated the relationship between character imageability and word imageability. Specifically, higher semantic transparency amplified the positive linear effect of character concreteness on word concreteness (Fig. 23).

We also explored the relationship between character-level and word-level variables when word-level lexico-semantic variables were not controlled and found a similar pattern of results for all variables except that neither first-character semantic transparency nor second-character semantic transparency moderated the relationship between character familiarity and word familiarity.

## Discussion

The present study aims to norm the ratings of valence and arousal, two core dimensions of emotions (e.g., Russell, 2003), of 3971 Chinese characters in traditional script and investigate the relationships among their affective and lexico-semantic variables. Specifically, we conducted a normed rating study for valence and arousal of the base characters from the large pool of 25,281 two-character words compiled by Tse et al.’s (2017, 2023) Chinese Lexicon Project. Both the mean (intensity) and standard deviation (ambiguity) of these normed affective variables are made freely accessible to the research communities (https://osf.io/kh4yx), following our previous practice (e.g., Chan & Tse, 2024; Tse et al., 2023). Using these normed data and those of previous studies (Chan & Tse, 2024; Su et al., 2022), we examined the relationships among affective and lexico-semantic variables of the characters, including their ambiguity measures, as well as the relationships across word-level and character-level variables, while controlling for other lexico-semantic variables. The key findings are summarized and discussed as follows.

### Examining inter-relationships among lexical variables of Chinese characters

We compared the inter-relationships among affective and lexico-semantic variables (valence, arousal, concreteness, imageability, and familiarity) within the pool of characters to those previously reported for simplified Chinese characters (e.g., Xue et al., 2024; Peng et al., 2024) and for its corresponding word pool (Chan & Tse, 2024), thereby exploring whether there could be any potential differences in the pattern between the two scripts.

Regarding the affective variables, we obtained an asymmetric valence–arousal relationship, where extremely negative characters are more arousing than extremely positive characters (Fig. 2). This pattern is consistent with findings for two-character words, where similar quadratic relationship was observed (e.g., Chan & Tse, 2024). Another nonlinear relationship we found is between valence and AoA, where extremely negative characters are acquired later in life than extremely positive characters (Fig. 6). This partially aligns with both Peng et al. (2024) and Xue et al. (2024) where negative correlation was observed. We, however, did not replicate Peng et al.’s weakly positive valence–familiarity relationship or Xue et al.’s weakly positive valence-imageability relationship (only for some subsets of characters), indicating that valence is not associated with familiarity or imageability (Figs. 3 and 5). Further, we found a positive arousal–AoA relationship, in contrast to the null relationship in Xue et al. The discrepancies between our study and previous studies may arise from the difference in analytic methods. While some studies relied on correlational analyses (e.g., Xue et al.), we ran regression analyses controlling for extraneous lexico-semantic variables (see, e.g., Table 2). The other findings of affective variables are in line with Chan and Tse’s (2024) findings on word variables: positive arousal–familiarity and arousal–imageability relationships and negative valence–concreteness and arousal–concreteness relationships. The consistencies of these relationships between words and characters suggests that the mechanisms under these inter-relationships among both affective and lexico-semantic variables operates similarly whether the unit is a character or a two-character word.

For the lexico-semantic variables, we replicated the positive imageability–familiarity and imageability–concreteness relationships and the negative AoA–concreteness and AoA–familiarity relationships (Liu et al., 2007; Wang et al., 2019; Su et al., 2022). However, the absence of AoA–imageability relationship and negative familiarity–concreteness relationship contradict the moderate positive familiarity–concreteness relationship (.470 in Liu et al., 2007;.460 in Su et al., 2022) and moderate negative AoA–imageability relationship (–.523 in Liu et al., 2007; – 0.491 in Su et al., 2022). It is noteworthy that we did find a positive trend of familiarity–concreteness relationship (see Fig. 4), as in previous studies, but this became negative when extraneous variables were strictly controlled. From the statistical point of view, this suppression effect suggests that the positive correlation between familiarity and concreteness observed in previous studies might be partially driven by the effect of some lexical variables, such as arousal, on both familiarity and concreteness. In other words, the influence of these variables, which were not controlled in prior works, might “mask” the genuine negative relationship by contributing positive covariance to the concreteness–familiarity association.

### Characterizing ambiguity measures

In addition to examining the intensity of various lexico-semantic and affective variables; that is, the average ratings across raters, we also examined their ambiguity, which to our knowledge has never been investigated in Chinese characters. In the present study, we examine the ambiguity measures in Chinese characters and compare the findings with those previously observed in two-character words (Chan & Tse, 2024), including the relationships between valence ambiguity and other lexico-semantic variables. We also explore how valence ambiguity and arousal ambiguity moderate the valence–arousal relationship and test whether the quadratic relationships between intensity (mean ratings) and ambiguity (standard deviation of ratings across raters), which were reported for valence, arousal, concreteness, and imageability, could be extended to the character level.

While Chan and Tse (2024) reported that valence, arousal, concreteness, imageability, and familiarly were correlated with valence ambiguity in two-character words, we found that only arousal, but not other variables, was associated with valence ambiguity. This shows that characters with higher arousal (e.g., 愛-love) might be more likely associated with more diverse valence ratings, depending on the raters’ idiosyncratic experience (e.g., some associate 愛 with exciting love experience, whereas others associate that with depressive breakup episodes). Nevertheless, it is not clear why valence ambiguity is not associated with other lexico-semantic or affective variables in characters.

Contrary to Chan and Tse’s (2024) findings that both valence ambiguity and arousal ambiguity moderated the quadratic valence–arousal relationship in two-character words, we only found the moderation effect of arousal ambiguity on the valence–arousal relationship. The valence–arousal relationship typically follows a U-shaped pattern under low arousal ambiguity, where extreme valence leads to heightened arousal. As arousal ambiguity increases, the relationship reverses into an inverted U-shape function, with characters with extreme valence being associated with lower arousal (Fig. 12). This pattern did not occur when valence ambiguity was examined as a moderating variable. The U-shaped valence–arousal relationship was consistent across different levels of valence ambiguity (Fig. 11). These findings were partially consistent with Brainerd’s (2021a, b) result that the increase in arousal ambiguity decreases the valence–arousal relationship.

All intensity–ambiguity relationships for lexico-semantic variables showed inverted U-shaped relationships, where characters with extreme variable values exhibit less variability than characters in mid-range. These replicated Brainerd et al. (2021a, b) and Chan and Tse’s (2024) quadratic intensity–ambiguity relationships for valence, arousal, familiarity, and concreteness. In the present study, we further extend this to AoA. The quadratic relationships are asymmetric in valence, arousal, familiarity, and AoA, and are symmetric in concreteness and imageability. Thus, for different variables, extreme values on the two ends are associated with their variability differently. This may arise from how tightly the two sets of variables are associated with universal norms, where the interpretation of the variables with asymmetric pattern (valence, arousal, familiarity, and AoA) could be influenced by cultural or personal experience. On the other hand, the interpretation of the variables with symmetric pattern (concreteness and imageability) may be influenced by sensory experiences, which provide consistent anchoring point about mid-concreteness and mid-imageability, as well as their distance to high-/low-concreteness and imageability. Our pattern of results is generally in line with those reported by Chan and Tse (2024) for two-character words, except that they found a U-shaped valence-valence ambiguity relationship, where negative and positive words are more ambiguous than neutral words.

### Cross-level analyses of character–word relationships

We conducted cross-level analyses to explore how character-level variables are associated with word-level variables. This includes examining the moderating role of semantic transparency, the degree of semantic relatedness between a word and its constituent characters (see Tse et al., 2017), and assessing the relative contributions of the first vs. second character to the overall lexical characteristics of a two-character word. Our results revealed that character-level variables are positively associated with the corresponding word-level variables, and this applies to both first and second characters. The relationships between character variables and word variables were generally stronger in first characters than in second characters for all variables. These findings suggest that valence, arousal, familiarity, concreteness, and imageability at the word level are more associated with those at the level of first character (vs. second character). However, the extent to how much stronger the first character-word variable association is than those of second character varies across different lexico-semantic variables. The difference was the largest for valence (.091), following arousal (.016) and imageability (.010), with familiarity (.009) and concreteness (.008) showcasing the smallest differences. This highlights the importance of controlling for the character-level variables even when studies aim only at investigating the word-level variables of Chinese words (see Tse & Yap, 2018, as an example). Future research should compare the predictive power of lexico-semantic variables at the word level, as well as for the first and second characters, in influencing performance on typical lexical processing tasks, such as lexical decision and speeded naming.

For most lexico-semantic and affective variables in the present study (valence, arousal, concreteness, and imageability), the semantic transparency of both first and second characters was found to amplify the positive linear relationship between character-level variables and word-level variables, with the only exception for the first-character’s and second-character’s familiarity. The character and word variables are more strongly associated for words with semantically transparent characters than those with semantically opaque characters. This is consistent with previous works (e.g., Myers et al., 2004; Tse & Yap, 2018; Tse et al., 2024) that semantic transparency could moderate the relationship between character-level and word-level representations. Future research should further investigate why the first-character familiarity is similarly associated with the word familiarity regardless of the semantic transparency. According to Balota and Chumbley (1984), character/word familiarity could be rated based on orthography and phonology, which may thus not necessarily capture only the character/word meaning to the same extent as the other variables being examined in the present study.

## Future directions

Given the interplay between the character-level and word-level representation in Chinese lexicon, the impact of lexical variables on lexical decision or naming performance could be attributed to the combined semantic or affective information of the word and individual characters. Focusing on the character level, if a character is high in arousal or strong in valence and is embedded in a two-character word, it can facilitate or interfere with lexical processing depending on task demands. For instance, high-arousal characters may accelerate the detection of affective words but could slow down the recognition if the surrounding context (i.e., another character or the whole word) is incongruent. These should be tested by examining the potential interaction of character-level and word-level variables (e.g., character frequency × word frequency) on the lexical processing performance, as reflected by lexical decision or speeded naming in future studies.

Another direction is to compare the present affective ratings and relationships with those established in other languages. For example, Ho et al. (2015) compared the normed ratings of ANEW and those in the Chinese-translated equivalent. While some words in two languages belong to the same valence category (e.g., despise), some words carry different valence information in different languages (e.g., crazy). Future research on investigating the similarities and differences among different languages and developing a comparative cross-linguistic framework is of great importance. In fact, future research should test whether the present pattern of relationships among affective and lexico-semantic variables, based on Chinese two-character words, could be generalized to English compound words (e.g., Kim et al., 2019), which share similar structures; that is, two constituents that are words on their own. This would further enhance our understanding of how constituents and words are represented at the semantic level of English lexicon.

Before concluding our study, it is important to highlight an inherent difference in Chinese characters and words that may complicate the interpretation of our findings. That is characters generally have more ambiguous meaning than words (e.g., Hoosain, 1991; Taft, 2003). Some characters are not very clear in meaning on their own, unless they are paired with another character (e.g., 生, 情, 烈). This might suggest that character lexico-semantic ratings are generally more varied than word lexico-semantic ratings (across raters); that is, the mean ambiguity of lexico-semantic variables might be larger for characters than for words. This is indeed the case when we compare the mean ambiguity of the characters in the present Table 1, with that of the words in Chan and Tse’s (2024) Table 1, except familiarity. To make sure the ambiguity measures at the character level might not complicate the interpretation of our findings, we re-ran all the above analyses without the character-level ambiguity measures being controlled. It is noteworthy that all patterns of results remained the same except that the valence–familiarity becomes significant (*p <.*001), and that semantic transparency moderates the relationship between the second character’s familiarity and word familiarity (*p <.*05) when character-level ambiguities are not controlled. This shows that our findings might not be compromised by potential general differences in ambiguities between characters and words.

We also reexamined the character-word level analyses using an alternative semantic ambiguity measure, perceived number of meanings (Chen et al., 2024b), defined as the number of meanings participants could think of for each given character, ranging from 0 to 5. This measure was normed on a set of simplified Chinese characters more highly overlapping with our present sample (*N* = 2376) than the other ambiguity measures (e.g., Chang & Lee, 2018; Chen et al., 2024a; Hsieh et al., 2024). Using perceived number of meanings as the moderator in cross-level analyses yielded a similar pattern of results, with the exception that it did not moderate the relationship between character and word valence in either the first or second character. It is worth noticing that as simplified Chinese characters could be more ambiguous (carry more meanings) than traditional Chinese characters (e.g.,发 could be 發/髮, 干 could be乾/幹), future studies should be cautious in comparing character ambiguity data across different scripts. In addition, future research should use other semantic ambiguity indices to further examine their moderating effects on the relationships between lexical variables at the character and word levels, thereby assessing the robustness and replicability of the present findings.

In the present study, Chinese characters are treated as isolated lexical units rather than as morphemes within two-character words. However, it is plausible that characters may function differently when embedded within words (e.g., Tse et al., 2024; Xue et al., 2024). Future studies should investigate how context-dependent ratings of Chinese characters (e.g., rating a character’s valence within transparent vs. opaque word contexts) differ from ratings obtained for standalone characters. Treating Chinese characters explicitly as morphological constituents within larger word units may have substantial implications for orthographic neighborhood (or morphological family) in the Chinese writing system (e.g., Li et al., 2015; Tsang et al., 2024; Tse et al., 2024). Finally, while our present semantic transparency measure relied upon participants’ subjective ratings (Tse et al., 2017), future studies could use distributional semantic models to generate transparency ratings, thus providing an objective validation of our findings.

## Conclusion

In the present study, we established the affective ratings (valence, arousal) of 3971 Chinese characters in traditional script, and demonstrated the relationships among these affective variables and other lexico-semantic variables. Building on the previous Chinese Lexicon Project (Tse et al., 2017, 2023; Chan & Tse, 2024), we also explored the cross-level relationships between character-level variables and word-level variables.

The findings revealed several important patterns. First, we replicated the quadratic valence–arousal relationship as shown in two-character words (Chan & Tse, 2024), such that negative and positive characters exhibit higher arousal than neutral characters. This curvilinear relationship could be moderated by arousal ambiguity, such that the increase in arousal ambiguity weaken the valence–arousal correlation, which was partially in line with previous findings (Brainerd et al., 2021a, b). Our findings on other relationships among lexico-semantic variables partially align with previous studies on simplified Chinese characters (Liu et al., 2007; Wang et al., 2019; Su et al., 2022; Xue et al., 2024) and two-character words (Chan & Tse, 2024), which could be attributed to a stricter control of extraneous variables in our regression analyses, as compared with mere bivariate correlation analyses in some studies (e.g., Xue et al., 2024). Second, we found quadratic intensity–ambiguity relationship for all variables included in the study (valence, arousal, familiarity, concreteness, imageability, and AoA), thus supporting the quadratic law proposed by Brainerd et al. (2021b). Third, we revealed that all character-level variables are positively associated with their corresponding word-level variables. This association applies to both first and second characters, and that the relationship is stronger in first characters than for second characters in all affective and lexico-semantic variables included in the study. However, the extent to how much the association in the first character is larger than that of the second character varied across affective and lexico-semantic variables. Semantic transparency is found to moderate cross-level variable relationships in all lexical variables but familiarity.

The present study normed the affective ratings (valence and arousal) of a large pool of 3971 traditional Chinese characters that is freely accessible to the researchers, providing invaluable resource for future psycholinguistic experiments such as matching specific lexical characteristics and investigation of other inter-relationships among lexical variables. By controlling for extraneous variables using multiple regression analyses, we extend previous works on Chinese characters and highlight the importance of incorporating various lexico-semantic variables in obtaining more accurate understanding of these relationships. Our cross-level analysis on the correlation between character-level and word-level variables, for the very first time, revealed the intertwining relationship among these lexico-semantic variables between Chinese character and word, which furthers our understanding about Chinese word-processing.

## Availability of data and materials & Code availability (Open Practices Statement)

The data, materials, and codes for analyses are freely accessible via https://osf.io/kh4yx. This study was not preregistered.

## References

[CR1] Balota, D. A., & Chumbley, J. I. (1984). Are lexical decisions a good measure of lexical access? The role of word frequency in the neglected decision stage. *Journal of Experimental Psychology Human Perception & Performance,**10*(3), 340–357. 10.1037/0096-1523.10.3.340 6242411 10.1037//0096-1523.10.3.340

[CR2] Bradley, M. M., & Lang, P. J. (1994). Measuring emotion: The self-assessment manikin and the semantic differential. *Journal of Behavior Therapy and Experimental Psychiatry,**25*(1), 49–59. 10.1016/0005-7916(94)90063-9 7962581 10.1016/0005-7916(94)90063-9

[CR3] Brainerd, C. J. (2018). The Emotional-Ambiguity Hypothesis: A large-scale test. *Psychological Science,**29*(10), 1706–1715. 10.1177/0956797618780353 30130163 10.1177/0956797618780353 PMC6180667

[CR4] Brainerd, C. J., Chang, M., & Bialer, D. M. (2021a). Emotional ambiguity and memory. *Journal of Experimental Psychology: General,**150*(8), 1476–1499. 10.1037/xge0001011 33332144 10.1037/xge0001011

[CR5] Brainerd, C. J., Chang, M., Bialer, D., & Toglia, M. P. (2021b). Semantic ambiguity and memory. *Journal of Memory and Language,**121*, 104286. 10.1016/j.jml.2021.104286

[CR6] Chan, Y.-L., & Tse, C.-S. (2024). Decoding the essence of two-character Chinese words: Unveiling valence, arousal, concreteness, familiarity, and imageability through word norming. *Behavior Research Methods,**56*(7), 7574–7601. 10.3758/s13428-024-02437-w 38750390 10.3758/s13428-024-02437-w PMC11362227

[CR7] Chang, M., & Brainerd, C. J. (2023). The recognition effects of attribute ambiguity. *Psychonomic Bulletin & Review,**30*(6), 2315–2327. 10.3758/s13423-023-02291-5 37131106 10.3758/s13423-023-02291-5

[CR8] Chang, Y. N., & Lee, C. Y. (2018). Semantic ambiguity effects on traditional Chinese character naming: A corpus-based approach. *Behavior Research Methods,**50*, 2292–2304. 10.3758/s13428-017-0993-4 29124717 10.3758/s13428-017-0993-4 PMC6267517

[CR9] Chang, Y., & Lee, C. (2020). Age of acquisition effects on traditional Chinese character naming and lexical decision. *Psychonomic Bulletin & Review,**27*(6), 1317–1324. 10.3758/s13423-020-01787-8 32789580 10.3758/s13423-020-01787-8 PMC7704508

[CR10] Chen, L., Xu, Y., & Perfetti, C. (2023a). A character-word dual function model of reading Chinese: Evidence from reading Chinese compounds. *Reading and Writing,**37*(9), 2429–2455. 10.1007/s11145-023-10478-4

[CR11] Chen, H., Xu, X., & Wang, T. (2023b). Assessing lexical ambiguity of simplified Chinese characters: Plurality and relatedness of character meanings. *Quarterly Journal of Experimental Psychology,**77*(4), 677–693. 10.1177/17470218231178787 10.1177/17470218231178787 37198743

[CR12] Chen, H., Xu, X., Li, H., Yu, X., Pan, R., & Zhang, Z. (2024a). A database of ambiguous Chinese characters with measures for meaning dominance and meaning balance. *Applied Psycholinguistics,**45*(4), 695–716. 10.1017/S0142716424000249

[CR13] Chen, H., Xu, X., & Wang, T. (2024b). Assessing lexical ambiguity of simplified Chinese characters: Plurality and relatedness of character meanings. *Quarterly Journal of Experimental Psychology,**77*(4), 677–693. 10.1177/17470218231178787 10.1177/17470218231178787 37198743

[CR14] Gagné, C. L., & Spalding, T. L. (2016). Effects of morphology and semantic transparency on typing latencies in English compound and pseudocompound words. *Journal of Experimental Psychology Learning Memory and Cognition,**42*(9), 1489–1495. 10.1037/xlm0000258 26889686 10.1037/xlm0000258

[CR15] Gong, Y., Lyu, B., & Gao, X. (2018). Research on teaching Chinese as a second or foreign language in and outside mainland China: A bibliometric analysis. *The Asia-Pacific Education Researcher,**27*(4), 277–289. 10.1007/s40299-018-0385-2

[CR16] Han, Y., Huang, S., Lee, C., Kuo, W., & Cheng, S. (2014). The modulation of semantic transparency on the recognition memory for two-character Chinese words. *Memory & Cognition,**42*(8), 1315–1324. 10.3758/s13421-014-0430-1 24894986 10.3758/s13421-014-0430-1

[CR17] Ho, S. M. Y., Mak, C. W. Y., Yeung, D., Duan, W., Tang, S., Yeung, J. C., & Ching, R. (2015). Emotional valence, arousal, and threat ratings of 160 Chinese words among adolescents. *PLoS ONE,**10*(7), e0132294. 10.1371/journal.pone.0132294 26226604 10.1371/journal.pone.0132294 PMC4520557

[CR18] Hoosain, R. (1991). Cerebral lateralization of bilingual functions after handedness switch in childhood. *The Journal of Genetic Psychology,**152*(2), 263–268. 10.1080/00221325.1991.9914672 1895073 10.1080/00221325.1991.9914672

[CR19] Hsieh, C.-Y., Marelli, M., & Rastle, K. (2024). Beyond quantity of experience: Exploring the role of semantic consistency in Chinese character knowledge. *Journal of Experimental Psychology: Learning, Memory, and Cognition,**50*(5), 819–832. 10.1037/xlm0001294 37883046 10.1037/xlm0001294

[CR20] Institute of Language Teaching and Research. (1984). *A frequency dictionary of modern Chinese*. Beijing Language Institute Press.

[CR21] Katz, L., & Frost, R. (1992). The reading process is different for different orthographies: The orthographic depth hypothesis. *Advances in Psychology,**94*, 67–84. 10.1016/s0166-4115(08)62789-2

[CR22] Kim, S. Y., Yap, M. J., & Goh, W. D. (2019). The role of semantic transparency in visual word recognition of compound words: A megastudy approach. *Behavior Research Methods,**51*, 2722–2732. 10.3758/s13428-018-1143-3 30291593 10.3758/s13428-018-1143-3

[CR23] Law, S.-P., Weekes, B. S., Wong, W., & Chiu, K. (2009). Reading aloud pseudo-characters by individuals with acquired dyslexia: Evidence for lexically-mediated processes in reading Chinese. *Language and Cognitive Processing,**24*, 983–1008. 10.1080/01690960802193696

[CR24] Li, M.-F., Lin, W.-C., Chou, T.-L., Yang, F.-L., & Wu, J.-T. (2015). The role of orthographic neighborhood size effects in Chinese word recognition. *Journal of Psycholinguistic Research,**44*(3), 219–236. 10.1007/s10936-014-9340-4 25451553 10.1007/s10936-014-9340-4

[CR25] Li, T., Wang, Y., Tong, X., & McBride, C. (2016). A developmental study of Chinese children’s word and character reading. *Journal of Psycholinguistic Research,**46*(1), 141–155. 10.1007/s10936-016-9429-z 10.1007/s10936-016-9429-z 27059992

[CR26] Liu, Y., Shu, H., & Li, P. (2007). Word naming and psycholinguistic norms: Chinese. *Behavior Research Methods,**39*(2), 192–198. 10.3758/bf03193147 17695344 10.3758/bf03193147

[CR27] Mattek, A. M., Wolford, G. L., & Whalen, P. J. (2017). A mathematical model captures the structure of subjective affect. *Perspectives on Psychological Science,**12*(3), 508–526. 10.1177/1745691616685863 28544868 10.1177/1745691616685863 PMC5445940

[CR28] Montefinese, M., Ambrosini, E., Fairfield, B., & Mammarella, N. (2014). The adaptation of the Affective Norms for English Words (ANEW) for Italian. *Behavior Research Methods,**46*(3), 887–903. 10.3758/s13428-013-0405-3 24150921 10.3758/s13428-013-0405-3

[CR29] Myers, J. (2010). Chinese as a natural experiment. *The Mental Lexicon,**5*(3), 421–435. 10.1075/ml.5.3.09mye

[CR30] Myers, J., Libben, G.,& Derwing, B. (2004). *The nature of transparency effects in Chinese compound processing*. Poster presented at the Fourth International Conference on the Mental Lexicon, Windsor.

[CR31] Packard, J. L. (2000). *The morphology of Chinese: A linguistic and cognitive approach*. Cambridge University Press. 10.1017/cbo9780511486821

[CR32] Peirce, J., Gray, J. R., Simpson, S., MacAskill, M., Höchenberger, R., Sogo, H., Kastman, E., & Lindeløv, J. K. (2019). PsychoPy2: Experiments in behavior made easy. *Behavior Research Methods,**51*(1), 195–203. 10.3758/s13428-018-01193-y 30734206 10.3758/s13428-018-01193-y PMC6420413

[CR33] Peng, D. L., Liu, Y., & Wang, C. (1999). How is access representation organized? The relation of polymorphemic words and their morphemes in Chinese. In J. Wang, A. W. Inhoff, & H.-C. Chen (Eds.), *Reading Chinese script: A cognitive analysis* (pp. 65–89). Lawrence Erlbaum. 10.4324/9781410601483-8

[CR34] Peng, C., Xu, X., & Bao, Z. (2024). Sentiment annotations for 3827 simplified Chinese characters. *Behavior Research Methods,**56*(2), 651–666. 10.3758/s13428-023-02068-7 36754941 10.3758/s13428-023-02068-7

[CR35] Perfetti, C. A., Zhang, S., & Berent, I. (1992). Reading in English and Chinese: Evidence for a “universal” phonological principle. *Advances in Psychology,**94*, 227–248. 10.1016/s0166-4115(08)62798-3

[CR36] Russell, J. A. (2003). Core affect and the psychological construction of emotion. *Psychological Review 110*(1), 145–172. 10.1037/0033-295X.110.1.145 10.1037/0033-295x.110.1.145 12529060

[CR37] Share, D. L. (2008). On the Anglocentricities of current reading research and practice: The perils of overreliance on an “outlier” orthography. *Psychological Bulletin,**134*(4), 584–615. 10.1037/0033-2909.134.4.584 18605821 10.1037/0033-2909.134.4.584

[CR38] Su, I. F., Yum, Y. N., & Lau, D. K. (2022). Hong Kong Chinese character psycholinguistic norms: Ratings of 4376 single Chinese characters on semantic radical transparency, age-of-acquisition, familiarity, imageability, and concreteness. *Behavior Research Methods,**55*(6), 2989–3008. 10.3758/s13428-022-01928-y 36002627 10.3758/s13428-022-01928-y PMC10558066

[CR39] Taft, M. (2003). Morphological representation as a correlation between form and meaning. In Neuropsychology and cognition (pp. 113–137). 10.1007/978-1-4757-3720-2_6

[CR40] Taft, M., Liu, Y., & Zhu, X. (1999). Morphemic processing in reading Chinese. In J. Wang, A. Inhoff, & H. C. Chen (Eds.), *Reading Chinese script: A cognitive analysis* (pp. 91–113). Lawrence Erlbaum. 10.4324/9781410601483-9

[CR41] Tan, L. H., & Perfetti, C. A. (1999). Phonological activation in visual identification of Chinese two-character words. *Journal of Experimental Psychology Learning Memory and Cognition,**25*(2), 382–393. 10.1037/0278-7393.25.2.382

[CR42] Tsang, Y. K., Zou, Y., Wang, J., & Wong, A.W.-K. (2024). Rethinking orthographic neighbor in Chinese two–character word recognition: Insights from a megastudy. *Psychonomic Bulletin & Review,**31*, 1588–1595. 10.3758/s13423-023-02434-8 38169040 10.3758/s13423-023-02434-8

[CR43] Tse, C.-S., & Yap, M. J. (2018). The role of lexical variables in the visual recognition of two-character Chinese compound words: A megastudy analysis. *Quarterly Journal of Experimental Psychology,**71*(9), 2022–2038. 10.1177/1747021817738965 10.1177/1747021817738965 30117382

[CR44] Tse, C.-S., Yap, M. J., Chan, Y.-L., Sze, W. P., Shaoul, C., & Lin, D. (2017). The Chinese Lexicon Project: A megastudy of lexical decision performance for 25,000+ traditional Chinese two-character compound words. *Behavior Research Methods,**49*(4), 1503–1519. 10.3758/s13428-016-0810-5 27734329 10.3758/s13428-016-0810-5

[CR45] Tse, C.-S., Chan, Y.-L., Yap, M. J., & Tsang, H. C. (2023). The Chinese Lexicon Project II: A megastudy of speeded naming performance for 25,000+ traditional Chinese two-character words. *Behavior Research Methods,**55*(8), 4382–4402. 10.3758/s13428-022-02022-z 36443581 10.3758/s13428-022-02022-z PMC9707223

[CR46] Tse, C.-S., Yap, M. J., & Chan, Y.-L. (2024). Neighborhood in Chinese lexicon: A megastudy analysis of lexical decision and naming of two-character Chinese words. *Journal of Experimental Psychology: Learning, Memory, and Cognition,**50*(9), 1489–1515. 10.1037/xlm0001357 39052394 10.1037/xlm0001357

[CR47] Tsung, L., & Cruickshank, K. (2011). Emerging trends and issues in teaching and learning Chinese. In Linda Tsung & Ken Cruickshank (Eds.), *Teaching and learning Chinese in global contexts: Multimodality and literacy in the new media age* (pp. 1–10). Continuum. 10.5040/9781474212373.ch-001

[CR48] Wang, H. (2011). *Single Chinese character-based study on modern Chinese morphology*. The Commercial Press.

[CR49] Wang, R., Huang, S., Zhou, Y., & Cai, Z. G. (2019). Chinese character handwriting: A large-scale behavioral study and a database. *Behavior Research Methods,**52*(1), 82–96. 10.3758/s13428-019-01206-4 10.3758/s13428-019-01206-4 30805862

[CR50] Warriner, A. B., Kuperman, V., & Brysbaert, M. (2013). Norms of valence, arousal, and dominance for 13,915 English lemmas. *Behavior Research Methods,**45*(4), 1191–1207. 10.3758/s13428-012-0314-x 23404613 10.3758/s13428-012-0314-x

[CR51] Xue, L., Li, D., Song, D., & Ma, W. (2024). Similarities and differences between Chinese two-character words and their constituent characters in norm-feature correlations. *Current Psychology,**43*, 25389–25402. 10.1007/s12144-024-06212-0

[CR52] Yee, L. T. S. (2017). Valence, arousal, familiarity, concreteness, and imageability ratings for 292 two-character Chinese nouns in Cantonese speakers in Hong Kong. *PLoS ONE,**12*(3), e0174569. 10.1371/journal.pone.0174569 28346514 10.1371/journal.pone.0174569 PMC5367816

[CR53] Yin, B., & Snowden Rohsenow, J. (1994). *Modern Chinese characters*. Sinolingua.

[CR54] Yum, Y. N., Law, S.-P., Su, I.-F., Lau, K.-Y.D., & Mo, K. N. (2014). An ERP study of effects of regularity and consistency in delayed naming and lexicality judgment in a logographic writing system. *Frontiers in Psychology,**5*, 315. 10.3389/fpsyg.2014.00315 24782812 10.3389/fpsyg.2014.00315 PMC3995054

[CR55] Zhou, X., & Marslen-Wilson, W. (2000). Lexical representation of compound Words: Cross-linguistic evidence. *Psychologia: An International Journal of Psychology in the Orient,**43*(1), 47–66.

