# Correction: Unveiling the intensity–ambiguity relationships among affective and lexico-semantic variables in Chinese characters and the character–word relationships in Chinese two-character words

**DOI:** 10.3758/s13428-025-02795-z

**Published:** 2025-08-27

**Authors:** Xi Cheng, Chi-Shing Tse, Yuen-Lai Chan, Kai Yan Lau, Yen Na Yum

**Affiliations:** 1 Department of Educational Psychology, The Chinese University of Hong Kong, Hong Kong, China; 2 Centre for Learning Sciences and Technologies, The Chinese University of Hong Kong, Hong Kong, China; 3 Department of Psychology, Lingnan University, Hong Kong, Hong Kong, China; 4 Department of Chinese and Bilingual Studies, The Hong Kong Polytechnic University, Hong Kong, China; 5 Department of Special Education and Counselling, The Education University of Hong Kong, Hong Kong, China

**Correction: Behavior Research Methods (2025) 57:224**

10.3758/s13428-025-02753-9

The original online version of this article was revised: In the section Materials and procedure, the sentence: Word-level variables, including valence, arousal, familiarity, concreteness, imageability, and AoA, were retrieved from Chan and Tse (2024)....

Was changed to: Word-level variables, including valence, arousal, familiarity, concreteness, and imageability, were retrieved from Chan and Tse (2024)....

